# Thioether Oxidation Chemistry in Reactive Oxygen Species (ROS)-Sensitive Trigger Design: A Kinetic Analysis

**DOI:** 10.1021/acs.orglett.5c00747

**Published:** 2025-03-19

**Authors:** Ayatullah Gamal Abdelfattah, Shubham Bansal, Joanna Afokai Quaye, Shameer M. Kondengadan, Giovanni Gadda, Binghe Wang

**Affiliations:** Departments of Chemistry and Biology and Center for Diagnostics and Therapeutics, Georgia State University, Atlanta, Georgia 30301, United States

## Abstract

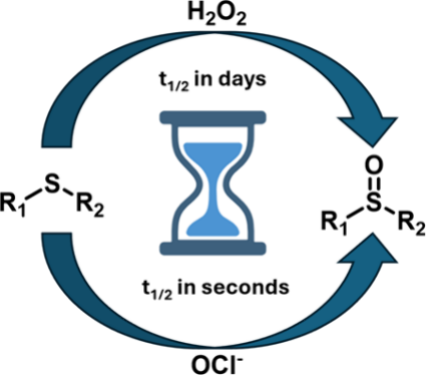

Thioether oxidation to sulfoxide by H_2_O_2_ has been widely reported as an ROS-sensitive trigger in drug delivery applications. Through a number of straightforward kinetic experiments with a series of aryl thioethers, we show that H_2_O_2_ oxidation under near-physiological conditions is expected to have half-lives on the scale of hundreds of hours at pathophysiologically relevant H_2_O_2_ concentrations. On the other hand, hypochlorite can oxidize thioethers at much faster rates with half-lives in the range of seconds to sulfoxide and minutes to sulfone under similar conditions. Such information means that hypochlorite likely plays a much more important role than H_2_O_2_ in activating thioether-based drug delivery systems.

Reactive oxygen species (ROS) play important roles in pathophysiological processes.^1−4^ A large number of pathological conditions are known to disrupt normal redox homeostasis, leading to over production of ROS.^5−10^ Therefore, there are widespread interests in ROS-sensitive drug delivery,^11−15^ through the use of an ROS-labile group for activation. Commonly used ROS-labile groups include the boronate group,^16−18^ sulfur/selenide/telluride ethers,^19−26^ and thioketals (Figure 1).^25,27−29^ Because ROS represents a group of commonly seen reactive species with widely different reactivity including H_2_O_2_, HOCl/OCl^–^, ONOO^–^, HO·, and O_2_^–^, there is a need to differentiate them to truly understand the roles of individual ROS for understanding their pathophysiological roles and for designing ROS-sensitive drug delivery^20,30−32^ and imaging systems.^33−35^ In doing so, reaction kinetics is a critical factor to consider.^36,37^

**Figure 1:**
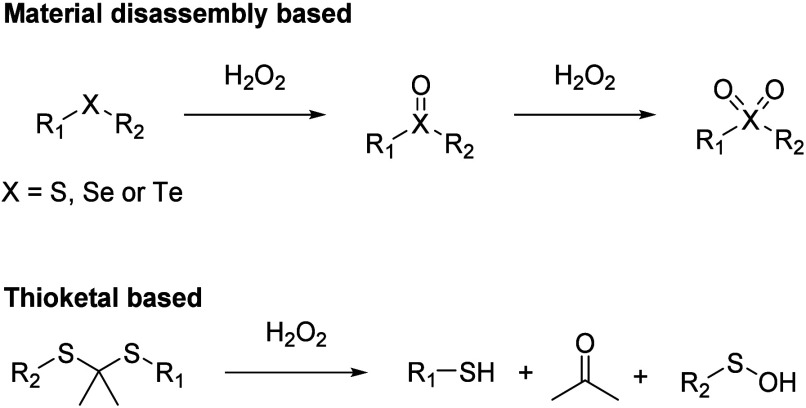
Proposed activation chemistry of thioether/thioketal-based drug delivery systems.

Among all the ROS-sensitive drug delivery systems, H_2_O_2_-sensitive triggers are the most widely studied because of the known high abundance of H_2_O_2_ (610 μM under certain pathological conditions vs 3 μM in the blood).^36,38^ Along this line, thioether oxidation has been reported as a H_2_O_2_-sensitive trigger for various applications.^23,25,39,40^

As background information, the second-order rate constant of methionine thioether oxidation is 2 × 10^–2^ M^–1^ s^–1^ by H_2_O_2_^41^ and 3.7 × 10^8^ M^–1^ s^–1^ by hypochlorite.^42,43^ Such information hints at the grossly overlooked roles of hypochlorite in the activation of thioether-based drug delivery systems, especially considering the fact that hypochlorite is the second most abundant ROS with concentration reaching 398 μM in neutrophils upon stimulation and 39 μM without any stimulation.^44^ Therefore, we were interested in doing some simple side-by-side comparisons of activation kinetics/half-life in order to gain insights into the roles each ROS (H_2_O_2_ and hypochlorite) may play in activating a thioether-based drug delivery system. Below, we describe our findings.

For ease of monitoring spectroscopic changes, we first designed a series of thiophenol ethers (thioanisole) for studying oxidation kinetics and for examining the electronic effects on the oxidation rate by H_2_O_2_ and hypochlorite. We also selected an aliphatic thioether analog, which has been reported to show fluorescent changes upon oxidation.^45^ As the first step, we needed to synthesize these compounds.

## Synthesis

**Scheme 1:**
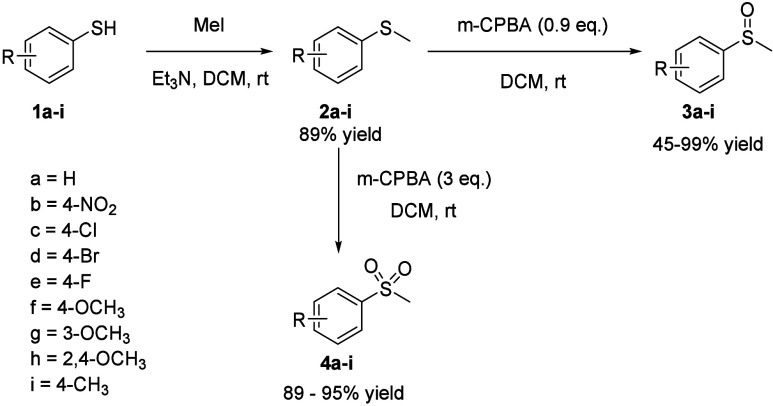
Synthesis of Thioethers and the Corresponding Sulfoxides or Sulfones

The synthesis of the designed compounds and the oxidation products is straightforward (Scheme 1) and described in detail in the SI. We included aromatic thioethers with both an electron-donating group (EDG, **2f**–**i**) or an electron-withdrawing group (EWG, **2b**–**e**) in order to understand the effect of substituent group on reaction kinetics. However, for studies using NaOCl, we only focused on analogs with an electron-withdrawing group (EWG, **2b**–**e**) because of the extremely fast reaction of the other analogs (see below). One aliphatic thioether analog **5** was also chosen and was synthesized by following reported procedures (Scheme S3).^45^

## Oxidation Kinetics by H_2_O_2_

**Figure 2:**
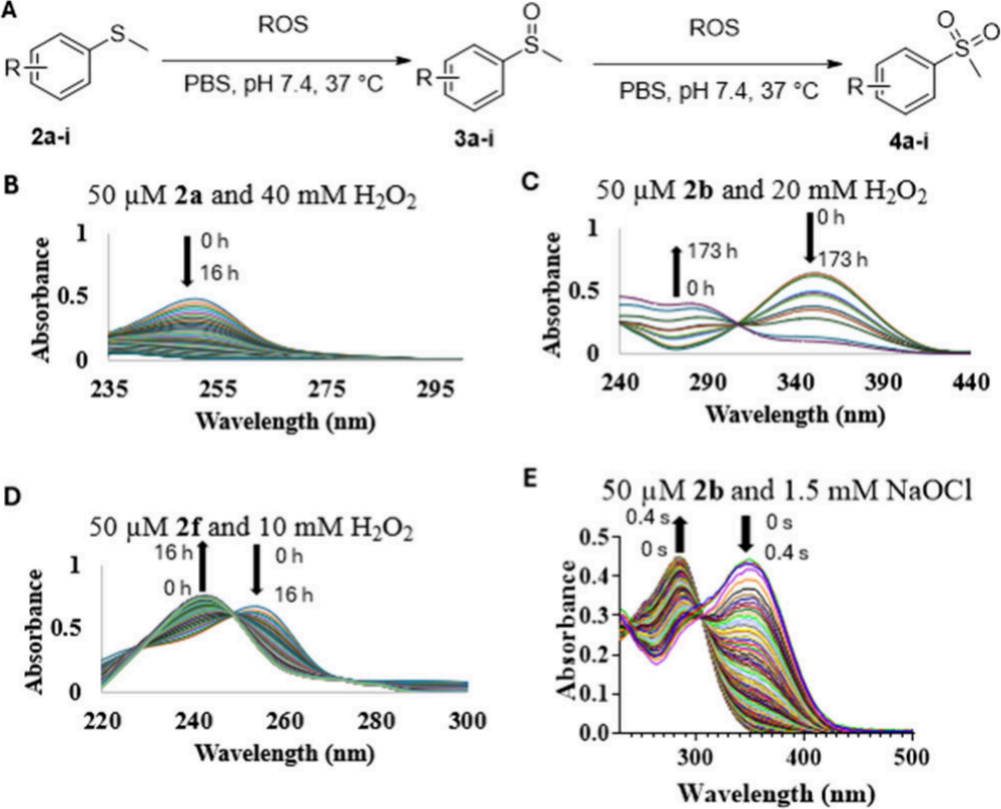
(A) Reaction scheme of thioether oxidation by ROS in PBS at pH 7.4 and 37 °C; (B–E) UV–vis spectral changes of thioether reaction with ROS at pH 7.4 and 37 °C.

The basic requirement of an ROS-sensitive group for drug delivery is its oxidation by the intended ROS with appropriate kinetics. Therefore, we first studied thioether oxidation by H_2_O_2_. Briefly, we first determined the pseudo first-order rate constant by using 50 μM thioether **2a** with H_2_O_2_ at different concentrations (10 to 40 mM) in PBS (containing 20% methanol) at pH 7.4 and 37 °C. Figure 2B shows the spectroscopic profile of the reaction. Following standard procedures, we plotted the pseudo-first order rate constant against H_2_O_2_ concentration, yielding a second-order rate constant of 2.53 ± 1.18 × 10^–3^ M^–1^ s^–1^ (Figure S1 and Table 1) and a calculated first half-life of ∼45 days at 100 μM each of the reactant. Obviously, this half-life is too long to be meaningful for biological applications. Based on the proposed reaction mechanism (Scheme S1),^46^ one would anticipate strong electronic effects by substituents on the aryl ring. To study this aspect, we next examined four analogs with an EWG (Scheme 1, **2b**–**e**). Figure 2 shows a set of representative time-dependent UV spectra for **2b** (Figure 2C). As expected, EWG groups decreased the reaction rate compared to thioanisole itself (**2a**) (Figures S2–S5). Their second-order rate constants are shown in Table 1.

**Table 1:**
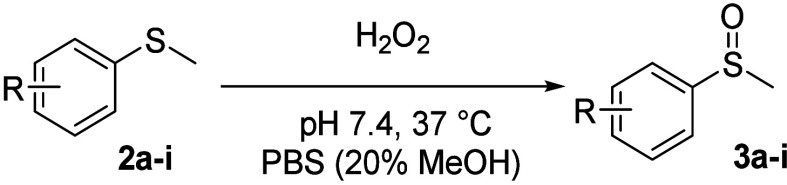
Reaction Rate of Thioether Oxidation by H_2_O_2_

compound #	compound	rate constant (M^–1^ s^–1^)	Hammett constant
**2a**	4-H	2.53 ± 1.18 × 10^–3^	0
**2b**	4-NO_2_	1.0 ± 0.0 × 10^–4^	0.778
**2c**	4-Cl	1.53 ± 0.15 × 10^–3^	0.227
**2d**	4-Br	1.40 ± 0.1 × 10^–3^	0.232
**2e**	4-F	1.57 ± 0.37 × 10^–3^	0.062
**2f**	4-OCH_3_	1.28 ± 0.31 × 10^–2^	–0.27
**2g**	3-OCH_3_	1.57 ± 0.15 × 10^–3^	0.12
**2h**	2,4-OCH_3_	3.57 ± 1.66 × 10^–3^	-
**2i**	4-CH_3_	4.33 ± 0.40 × 10^–3^	–0.17

In order to see whether we can increase the reaction rate to a point that would be practical for prodrug activation by H_2_O_2_, we studied four analogs with one or more EDG. Figure 2D shows a representative set of UV–vis profiles for **2f** (4-OCH_3_) as an example (others in the SI, Figures S6–S9). The second-order rate constant for the fastest thioether **2f** was determined to be 1.28 ± 0.31 × 10^–2^ M^–1^ s^–1^ (Table 1 and Figure 2), giving a half-life of around 75 h at 10 μM thioether **2f** and 200 μM H_2_O_2_. This is faster than the oxidation of **2a,** but still very slow for biologically relevant activation in cell culture or *in vivo*. The second-order rate constant of thioether **2i** (4-CH_3_) (Figure S6) falls between **2a** (-H) and **2f** (4-OCH_3_), as expected. For **2g** (3-OCH_3_), the reaction rate (Figure S8) was slower than the unsubstituted **2a.** This is easy to understand since a methoxy group is an EWG inductively at the meta-position. This is reflected in its positive Hammett constant value of 0.12.^47^ For the case of **2h,** the reaction rate (Figure S9) was between **2a** and **2f.** This is also easy to understand since ortho substitution can exert the known ortho effects, involving steric hindrance.^48−51^ All these are in line with what one would expect as shown in a Hammett plot (Figure S10).

**Figure 3:**
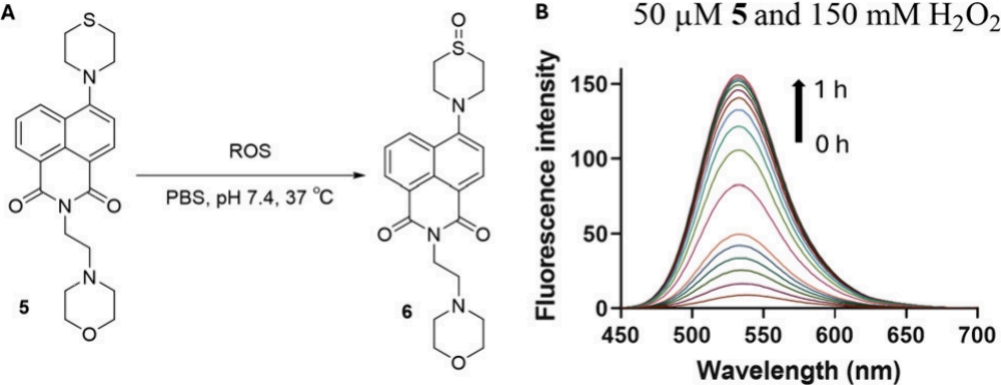
(A) Reaction scheme of thioether **5** oxidation by ROS in PBS at pH 7.4 and 37 °C; (B) UV–vis spectral changes of thioether **5** upon reaction with H_2_O_2_ at pH 7.4 and 37 °C.

Similarly, aliphatic analog **5** also showed slow reactivity with H_2_O_2_, as the second order rate constant was determined to be 6.7 ± 2.3× 10^–3^ M^–1^ s^–1^ (Table 1, Figure 3 and Figure S11) with a calculated first half-life of ∼17 days at 100 μM each, which is too long of a half-life for most meaningful biological applications.

Overall, thioether oxidation by H_2_O_2_ was found to be very slow even for the most reactive analog, **2f,** with the *t*_1/2_ being about 75 h at the higher end of biologically relevant H_2_O_2_ concentrations (∼200 μM).^36^ One important bottom-line message is clear that the rate of oxidation is likely too slow for meaningful applications in drug delivery under most circumstances.

## Oxidation of Thioethers by Hypochlorite

Hypochlorite is the second most abundant ROS and is known to oxidize amines and thioethers.^43,52^ Reactivity of hypochlorite also extends to the commonly used solvents such as DMSO.^37^ Unfortunately, the presence of hypochlorite is still not commonly considered in ROS-sensitive drug delivery designs. We are interested in studying the oxidation kinetics of the thioether group by NaOCl and how the kinetics can be tuned for desired applications.

We determined the reaction rate of thioether oxidation to sulfoxide and sulfoxide to sulfone,^37,53^ using stopped-flow for the experiments with NaOCl because of the anticipated fast reaction. Briefly, the pseudo first-order rate constant for the reaction of thioether **2a** at 50 μM with different concentrations of NaOCl (0.6–1.5 mM) was determined in PBS (containing 5% ACN) at pH 7.4 and 37 °C by monitoring the intensity decrease at 251 nm (**2a**) and increase at 230 nm (sulfoxide **3a**) (Figure S12). Complete consumption of thioether **2a** was observed within 10 ms (Figure S13), indicating a very fast reaction. Even at 25 and 10 °C, the reaction was still very fast, showing completion within 100 ms (Figure S13).

For the sulfoxide **3a** to sulfone reaction, the second-order rate constant was determined to be 102 ± 7.8 M^–1^ s^–1^ (Figure S14), which is slower than the oxidation to sulfoxide **3a** as discussed earlier. Overall, oxidation of thioether **2a** to sulfoxide **3a** and subsequent oxidation to sulfone **4a** by hypochlorite are both very fast. Next, we were interested in studying substituent effects on the oxidation of thioether by hypochlorite. Because the oxidation is already very fast with thioanisole, we only selected analogs with an EWG including 4-Cl, 4-Br, 4-F, and 4-NO_2_ for studies using similar procedures as described earlier (Figures S15–S20). The results are shown in Table 2 as well as Figures S15–S20.

**Table 2:**
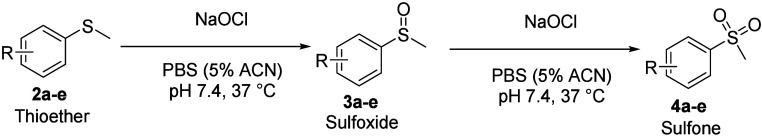
Reaction Rate of Thioether Oxidation by NaOCl

			thioether to sulfoxide	sulfoxide to sulfone	
compound #	compound	Hammett constant	rate constant (M^–1^ s^–1^)	rate constant (M^–1^ s^–1^)	first *t*_1/2_ at 10 μM each
**2/3/4a**	H	0	too fast, completed in 10 ms^a^	102 ± 7.8	16 min
**2/3/4b**	4-NO_2_	0.778	1.2 ± 0.08 × 10^4^	5.9 ± 1.3	5 h
**2/3/4c**	4-Cl	0.227	too fast, completed in 22 ms^a^	36 ± 3.2	46 min
**2/3/4d**	4-Br	0.232	too fast, completed in 12 ms^a^	9.9 ± 1.6	3 h
**2/3/4e**	4-F	0.062	too fast, completed in 20 ms^a^	77 ± 1.8	22 min

a Based on the reaction of 50 μM thioether and 0.6 mM NaOCl in PBS (containing 5% ACN) at pH 7.4 and 37 °C.

Among all the analogs with an EWG group, only the oxidation of 4-NO_2_ substituted thioether to sulfoxide (**2b**) by NaOCl was slow enough to allow for reaction rate constant determination, leading to a second-order rate constant of 1.2 ± 0.08 × 10^4^ M^–1^ s^–1^ (Figure 2E and Figure S19). Obviously, the second order rate constant of sulfoxide oxidation to sulfone was much slower than the first step, allowing for rate constant determination for all the analogs. For example, the rate constant for **3b** oxidation was determined to be 5.9 ± 1.3 M^–1^ s^–1^ (Figure S20). Such results are in agreement with a proposed reaction mechanism (Scheme S2) and the expected effects of aryl substituents.^53^ Overall, oxidation by NaOCl of the slowest thioether **2b** is 6 orders of magnitude faster than oxidation by H_2_O_2_ of the most reactive thioether **2f** analog. Hammett plot shows good ability to predict the reaction rate based on Hammett constant (Figure S21).

Similar to the thioanisole, aliphatic analog **5** also showed fast reaction with NaOCl, as the thioether was oxidized to sulfone within 2.2 ms (Figure S22). Such results are fully anticipated and are consistent with the findings of hypochlorite oxidation of aromatic thioethers. We should note that photodegradation was observed with this fluorescent compound upon prolonged exposure to laser light. However, we did not pursue this aspect because it does not impact the kinetic experiments and is beyond the scope of the current study.

Overall, all of the thioethers showed very fast reaction with NaOCl, much faster than the oxidation by H_2_O_2_ (Table 2). Even the slowest thioether oxidation (Table 2, Entry 2) by hypochlorite has a rate constant of 10^4^ M^–1^ s^–1^ (Table 2 and Figure S19), which is faster than many of click and biorthogonal reactions.^54^ Between the two most abundant ROS, the kinetic analysis indicates that thioether oxidation seems to be primarily due to the hypochlorite under therapeutically relevant conditions.

In ROS-sensitive drug delivery, the thioether moiety has been widely used as a H_2_O_2_-sensitive trigger. The oxidation of a thioether by H_2_O_2_ to sulfoxide and/or sulfone has been proposed as the triggering mechanism. We have demonstrated that such oxidation is expected to have long half-lives likely on the scale of hundreds of hours at 10 μM the thioether probe and 200 μM H_2_O_2_. Even the introduction of a strong EDG (4-OCH_3_) did not bring the reaction rate to the physiological relevant range. Thus, thioether oxidation by H_2_O_2_ is unlikely to be a meaningful event under biologically relevant conditions. On the other hand, we show that the second most abundant ROS, hypochlorite, can oxidize thioether to sulfoxide at much faster rates with half-lives in the range of seconds at 10 μM each of thioether and NaOCl. Even for the analogs with a strong EWG, the half-lives are still in the range of seconds at 10 μM each of thioether and NaOCl. Furthermore, hypochlorite can oxidize sulfoxide to sulfone under certain conditions. Such information means that thioether-based drug delivery systems are likely to be activated by hypochlorite, but not H_2_O_2_. We hope that the results will help others design their own experiments in ROS research.

## Data Availability

The data underlying this study are available in the published article and its Supporting Information.

## Supporting Information Available

Reaction rate determination studies, proposed reaction mechanisms of thioether oxidation to sulfoxides/sulfone by ROS, plots of second-order reaction rate vs Hammett constant, materials/methods, protocols for reaction rate determination studies, synthesis schemes and protocols, and characterization data and spectra including proton NMR, carbon NMR, and HRMS spectra (PDF)
