# Supplementary material for: Thioether Oxidation Chemistry in Reactive Oxygen Species (ROS)-Sensitive Trigger Design: A Kinetic Analysis
Source: Org Lett. 2025 Mar 19;27(12):3071–6. doi: 10.1021/acs.orglett.5c00747 (PMC11959603; doi:10.1021/acs.orglett.5c00747)
Supplement: Supplementary file 1 — ol5c00747_si_001.pdf [file ol5c00747_si_001.pdf]

Supporting Information

Thioether Oxidation Chemistry in Reactive Oxygen Species (ROS)-sensitive Trigger Design: A Kinetic Analysis

Ayatullah Gamal Abdelfattah,[#] Shubham Bansal,[#] Joanna Afokai Quaye, Shameer M. Kondengadan, Giovanni Gadda, and Binghe Wang*

Department of Chemistry and Center for Diagnostics and Therapeutics, Georgia State University, Atlanta, Georgia 30301 USA

[#]These authors contributed equally to this work.

*Address correspondence to
Dr. Binghe Wang
Regents Professor and Dr. Frank Hannah Chair
Georgia Research Alliance Eminent Scholar
Department of Chemistry
Georgia State University
Atlanta, Georgia 30301
USA
wang@gsu.edu
Phone: 404-413-5544
ORCID: 0000-00002-2200-5270

1. Oxidation of thioethers by hydrogen peroxide

1.2. Second order rate determination of thioether 2a oxidation by H_2O_2

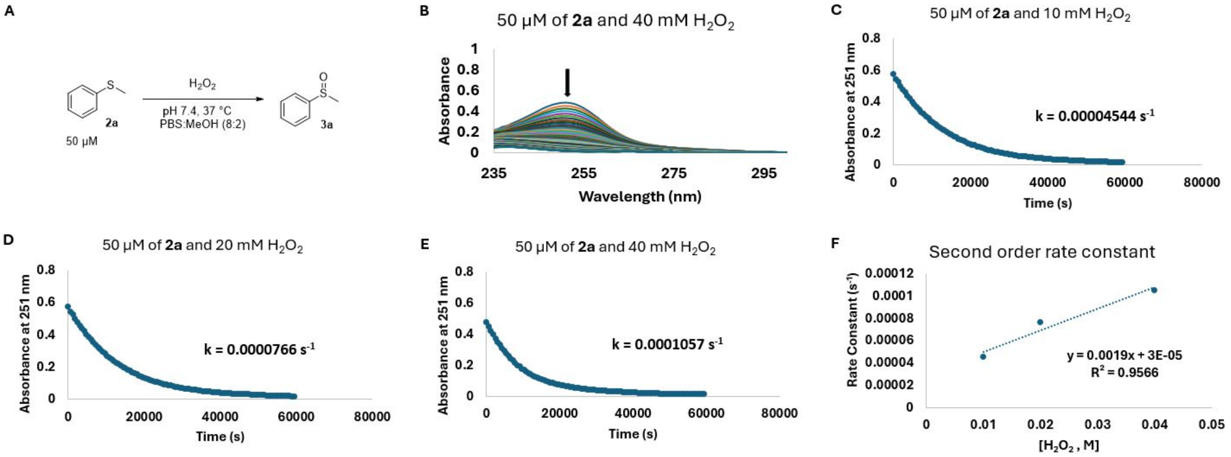

*Figure S1. (A) Reaction scheme of **2a** (thioanisole, 50 μM) oxidation by H_2O_2 in PBS at pH 7.4 & 37 °C (B) UV-Vis spectral changes with the reaction progression; (C) Reaction of **2a** with 10 mM H_2O_2. (D) Reaction of **2a** with 20 mM H_2O_2; (E) Reaction of **2a** with 40 mM H_2O_2; (F) Second-order rate determination of **2a** and H_2O_2. Figure represents a representative trail and average of three trials in shown in Table S1.*

*Table S1. Reaction rate of thioether **2a** oxidation by hydrogen peroxide*

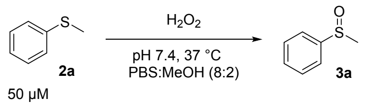

Trials	Pseudo-first order rate constant (s^{-1})			Second order rate constant ($M^{-1}\ s^{-1}$)
	10 mM H_2O_2	20 mM H_2O_2	40 mM H_2O_2	
1	0.00004544	0.0000766	0.0001057	0.0019
2	0.00004442	0.00007516	0.0001014	0.0018
3	0.00003338	0.0000766	0.0001523	0.0039

1.3. A proposed reaction mechanism of thioether oxidation to sulfoxides by H_2O_2

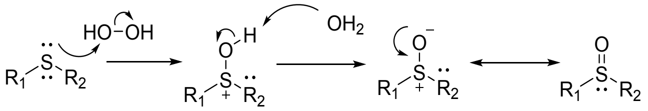

Scheme S1. A proposed reaction mechanism of thioether oxidation to sulfoxides by H_2O_2.

1.4. Second order rate determination of thioether 2b oxidation by H_2O_2

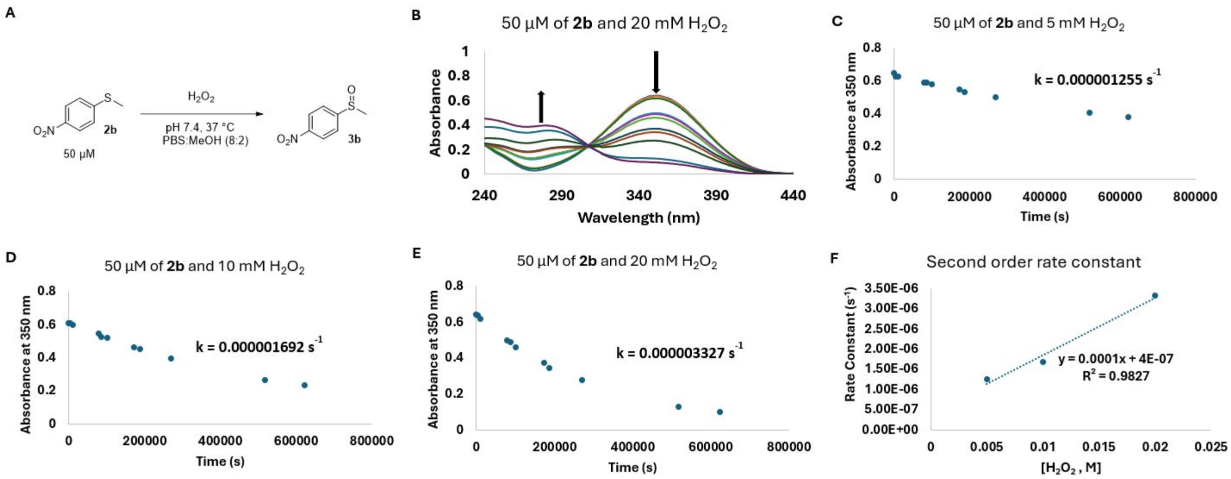

*Figure S2. (A) Reaction scheme of **2b** (4-nitrothioanisole, 50 µM) oxidation by H_2O_2 in PBS at pH 7.4 & 37 °C. (B) UV-Vis spectral changes showing the reaction progression; (C) Reaction of **2b** with 10 mM H_2O_2; (D) Reaction of **2b** with 20 mM H_2O_2; (E) Reaction of **2b** with 40 mM H_2O_2; (F) Second order rate determination of **2b** and H_2O_2. Figure represents a representative trail and average of trials is shown in Table S2.*

*Table S2. Reaction rate of thioether **2b** oxidation by hydrogen peroxide*

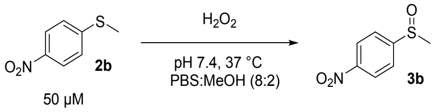

Trials	Pseudo-first order rate constant (s^{-1})			Second order rate constant (M^{-1} s^{-1})
	5 mM H_2O_2	10 mM H_2O_2	20 mM H_2O_2	
1	0.000001255	0.000001692	0.000003327	0.0001
2	0.000001704	0.00000253	0.000003712	0.0001

1.5. Second order rate determination of thioether 2c oxidation by H_2O_2

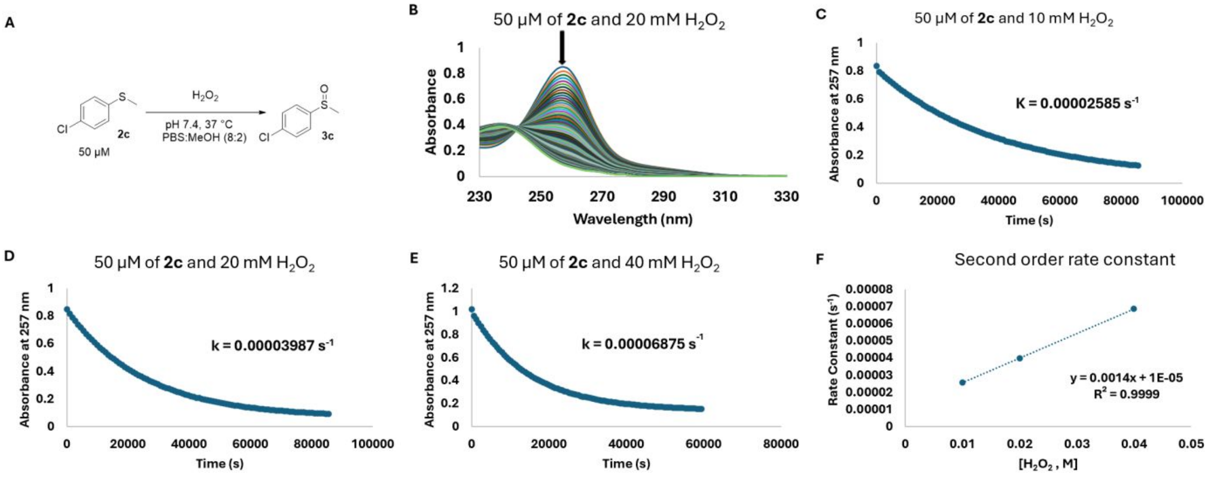

*Figure S3. (A) Reaction scheme of **2c** (50 µM) oxidation by H_2O_2 in PBS at pH 7.4 & 37 °C. (B) UV-Vis spectral changes showing the reaction progression; (C) Reaction of **2c** with 10 mM H_2O_2; (D) Reaction of **2c** with 20 mM H_2O_2; (E) Reaction of **2c** with 40 mM H_2O_2; (F) Second order rate determination of **2c** and H_2O_2. Figure represents a representative trail and average of trials is shown in Table S3.*

*Table S3. Reaction rate of thioether **2c** oxidation by hydrogen peroxide*

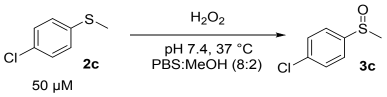

Trials	Pseudo-first order rate constant (s^{-1})			Second order rate constant (M^{-1} s^{-1})
	10 mM H_2O_2	20 mM H_2O_2	40 mM H_2O_2	
1	0.00002585	0.00003987	0.00006875	0.0014
2	0.00002375	0.00003965	0.00007419	0.0017
3	0.00003092	0.00003925	0.00007564	0.0015

1.6.Second order rate determination of thioether 2d oxidation by H_2O_2

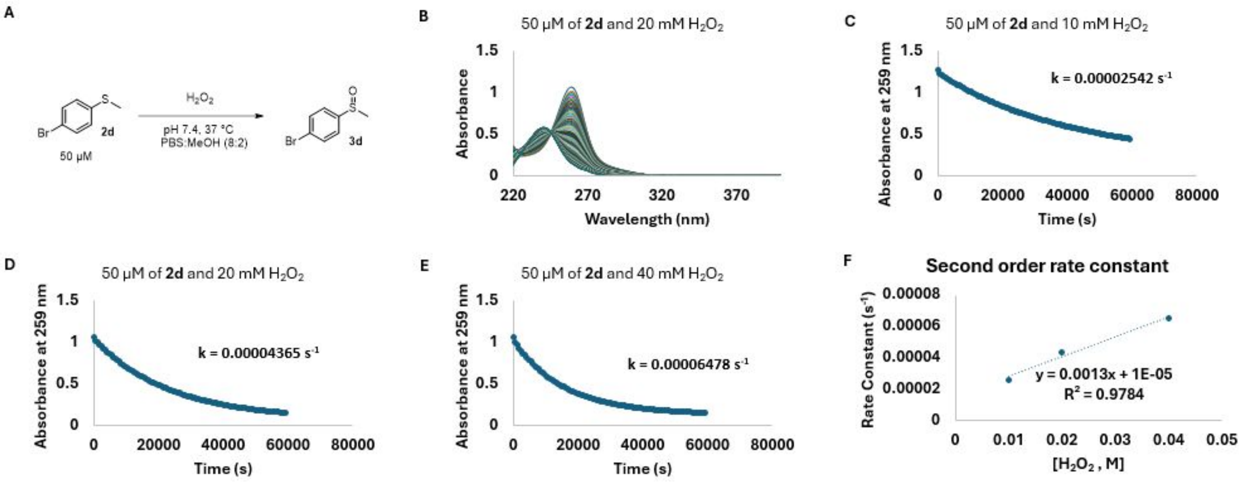

*Figure S4. (A) Reaction scheme of **2d** (50 μM) oxidation by H_2O_2 in PBS at pH 7.4 & 37 °C. (B) UV-Vis spectral changes showing the reaction progression; (C) Reaction of **2d** with 10 mM H_2O_2; (D) Reaction of **2d** with 20 mM H_2O_2; (E) Reaction of **2c** with 40 mM H_2O_2; (F) Second order rate determination of **2d** and H_2O_2. Figure represents a representative trail and average of trials is shown in Table S4.*

*Table S4. Reaction rate of thioether **2d** oxidation by hydrogen peroxide*

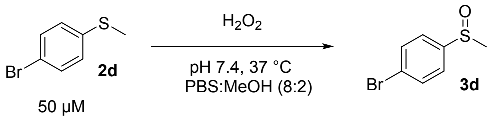

Trials	Pseudo-first order rate constant (s^{-1})			Second order rate constant (M^{-1} s^{-1})
	10 mM H_2O_2	20 mM H_2O_2	40 mM H_2O_2	
1	0.00002542	0.00004365	0.00006478	0.0013
2	0.00002532	0.00004037	0.00006863	0.0014
3	0.00002129	0.00003577	0.00006533	0.0015

1.7. Second order rate determination of thioether 2e oxidation by H_2O_2

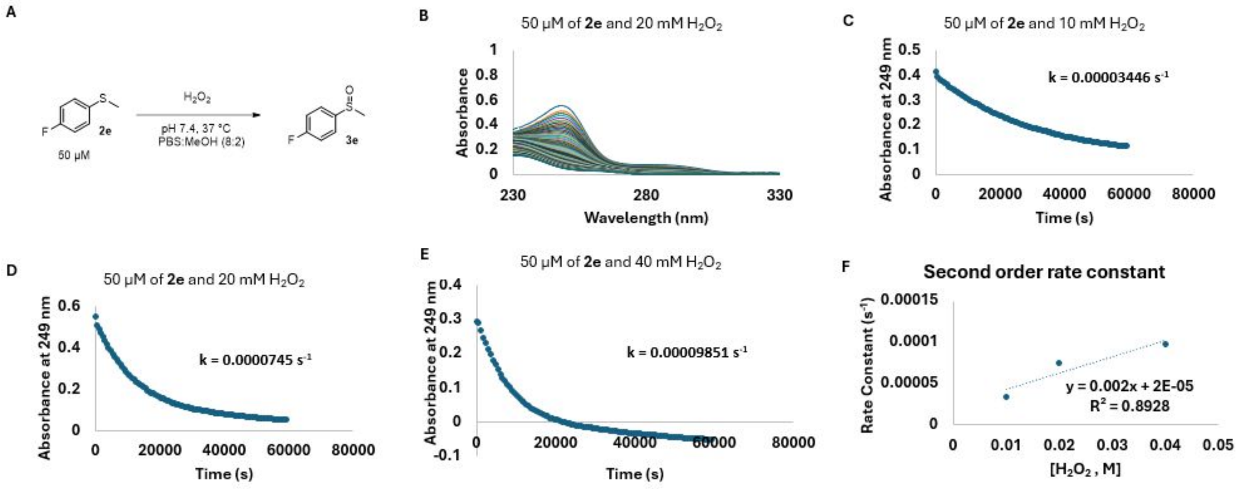

*Figure S5. (A) Reaction scheme of **2e** (50 μM) oxidation by H_2O_2 in PBS at pH 7.4 & 37 °C. (B) UV-Vis spectral changes showing the reaction progression; (C) Reaction of **2e** with 10 mM H_2O_2; (D) Reaction of **2e** with 20 mM H_2O_2; (E) Reaction of **2e** with 40 mM H_2O_2; (F) Second order rate determination of **2e** and H_2O_2. Figure represents a representative trail and average of trials is shown in Table S5.*

*Table S5. Reaction rate of thioether **2e** oxidation by hydrogen peroxide*

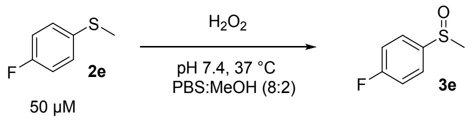

Trials	Pseudo-first order rate constant (s^{-1})			Second order rate constant (M^{-1} s^{-1})
	10 mM H_2O_2	20 mM H_2O_2	40 mM H_2O_2	
1	0.00003446	0.0000745	0.00009851	0.002
2	0.00004001	0.00006677	0.0000899	0.0016
3	0.00004177	0.00005144	0.00007351	0.0011

1.8. Second order rate determination of thioether 2i oxidation by H_2O_2

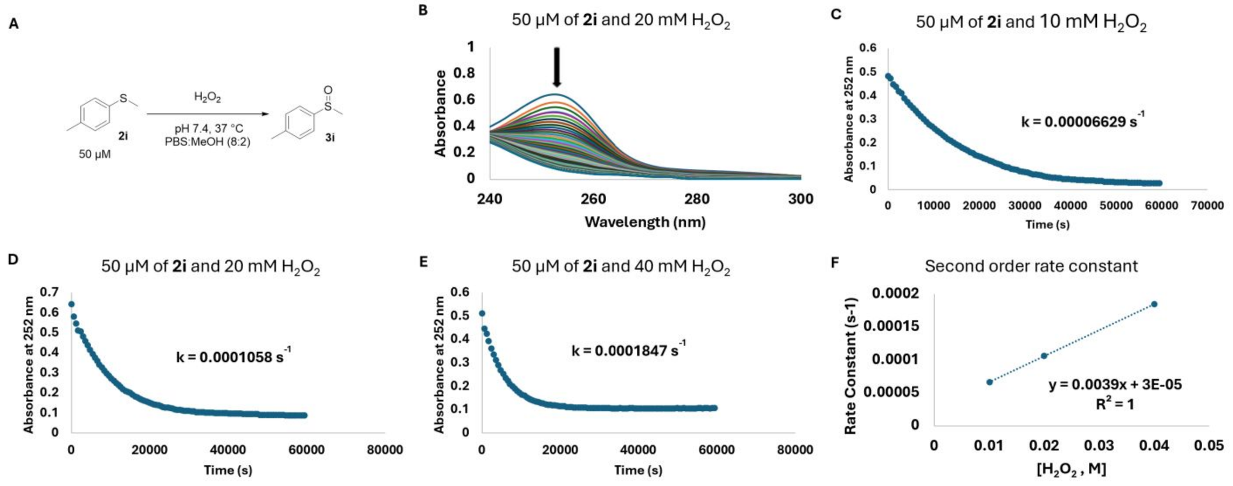

*Figure S6. (A) Reaction scheme of **2i** (50 µM) oxidation by H_2O_2 in PBS at pH 7.4 & 37 °C. (B) UV-Vis spectra's showing the reaction progress; (C) Reaction of **2i** with 10 mM H_2O_2. (E) Reaction of **2i** with 20 mM H_2O_2. (F) Reaction of **2i** with 40 mM H_2O_2. (F) Second-order rate determination of **2i** and H_2O_2. Figure represents a representative trail and average of three trials in shown in Table S6.*

*Table S6. Reaction rate of thioether **2i** oxidation by hydrogen peroxide*

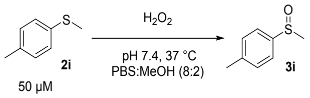

Trials	Pseudo-first order rate constant (s^{-1})			Second order rate constant (M^{-1} s^{-1})
	10 mM H_2O_2	20 mM H_2O_2	40 mM H_2O_2	
1	0.00006629	0.0001058	0.0001847	0.0039
2	0.00005196	0.0001035	0.0001925	0.0047
3	0.00005633	0.0001063	0.0001908	0.0044

1.9. Second order rate determination of thioether 2f oxidation by H_2O_2

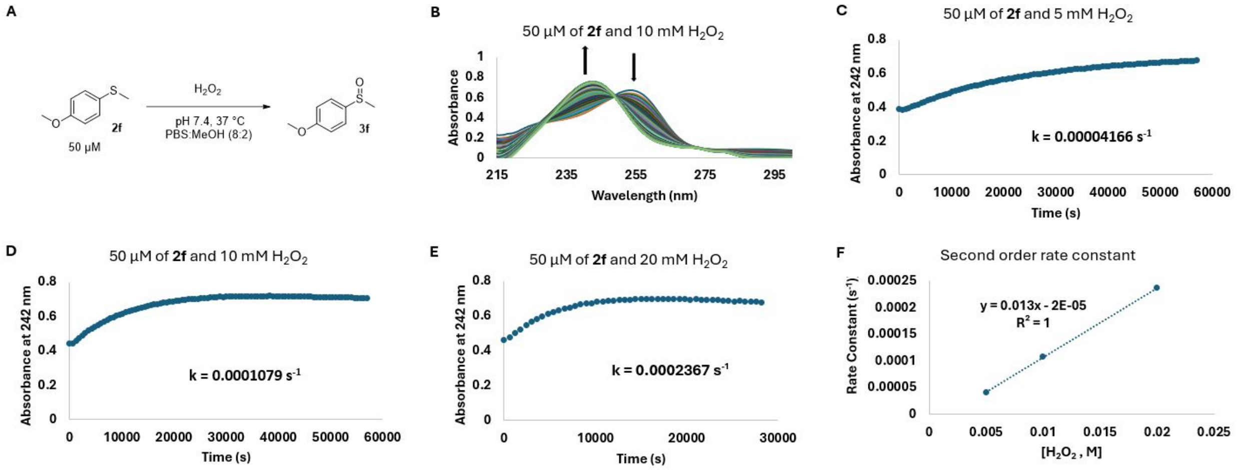

*Figure S7. (A) Reaction scheme of **2f** (4-methoxythioanisole, 50 μM) oxidation by H_2O_2 in PBS at pH 7.4 & 37 °C. (B) UV-Vis spectra's showing the reaction progress; (C) Reaction of **2f** with 10 mM H_2O_2. (E) Reaction of **2f** with 20 mM H_2O_2. (F) Reaction of **2f** with 40 mM H_2O_2. (F) Second-order rate determination of **2f** and H_2O_2. Figure represents a representative trail and average of three trials in shown in Figure S7.*

*Table S7. Reaction rate of thioether **2f** oxidation by hydrogen peroxide*

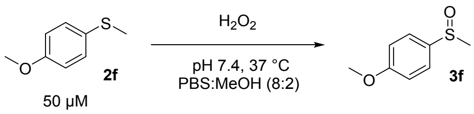

Trials	Pseudo-first order rate constant (s^{-1})			Second order rate constant (M^{-1} s^{-1})
	5 mM H_2O_2	10 mM H_2O_2	20 mM H_2O_2	
1	0.00005141	0.0001178	0.0002857	0.0158
2	0.00004166	0.0001079	0.0002367	0.013
3	0.00004815	0.0001326	0.0002008	0.0097

1.10. Second order rate determination of thioether 2g oxidation by H_2O_2

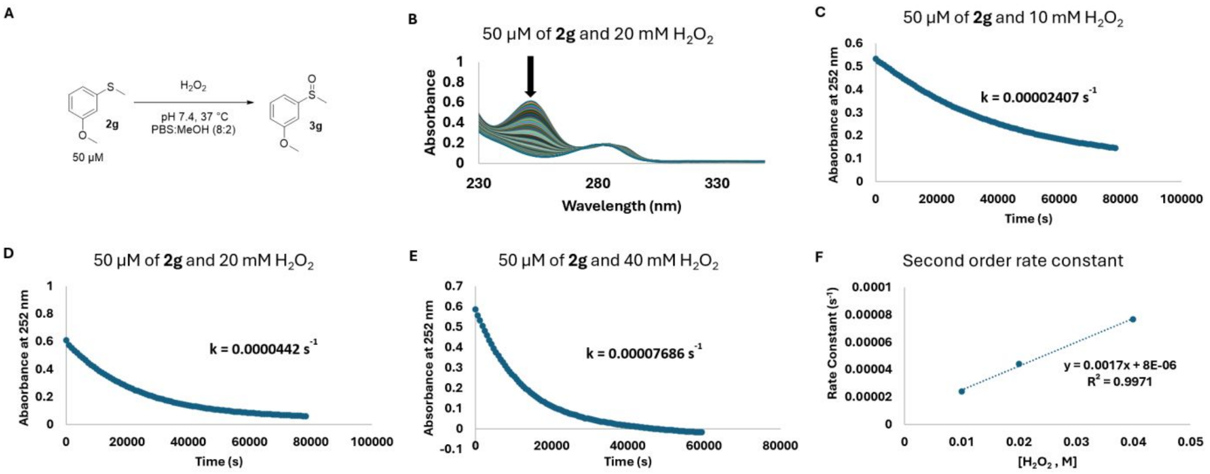

*Figure S8. (A) Reaction scheme of **2g** (50 µM) oxidation by H_2O_2 in PBS at pH 7.4 & 37 °C. (B) UV-Vis spectra's showing the reaction progress; (C) Reaction of **2g** with 10 mM H_2O_2. (E) Reaction of **2g** with 20 mM H_2O_2. (F) Reaction of **2g** with 40 mM H_2O_2. (F) Second-order rate determination of **2g** and H_2O_2. Figure represents a representative trail and average of three trials is shown in Table S8.*

*Table S8. Reaction rate of thioether **2g** oxidation by hydrogen peroxide*

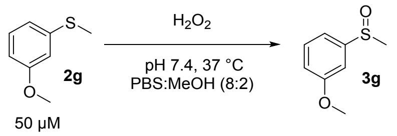

Trials	Pseudo-first order rate constant (s^{-1})			Second order rate constant ($M^{-1}\ s^{-1}$)
	10 mM H_2O_2	20 mM H_2O_2	40 mM H_2O_2	
1	0.00002325	0.00005297	0.00007405	0.0016
2	0.00002407	0.0000442	0.00007686	0.0017
3	0.00002675	0.00004434	0.0000685	0.0014

1.11. Second order rate determination of thioether 2h oxidation by H_2O_2

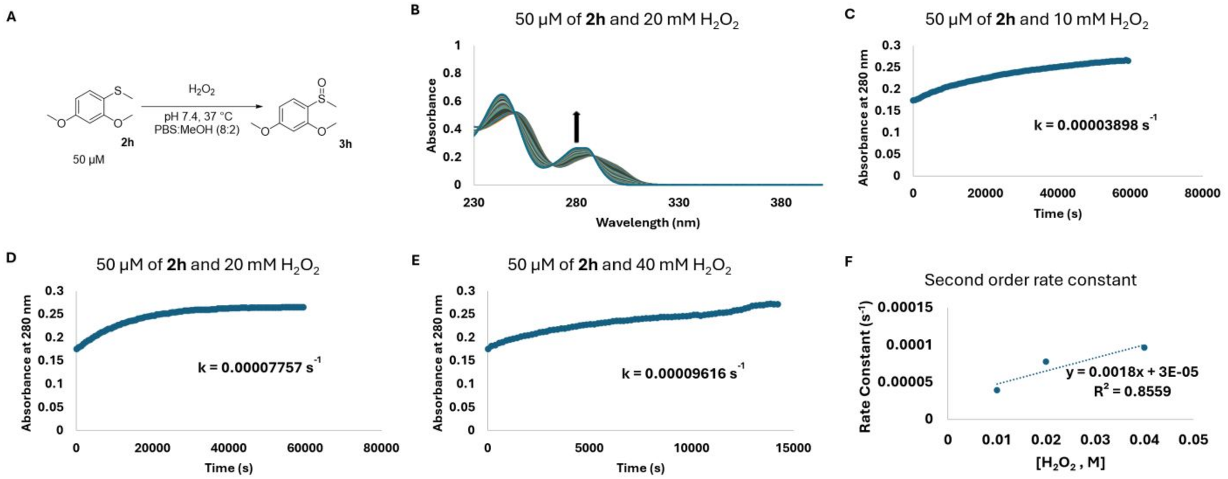

*Figure S9. (A) Reaction scheme of **2h** (50 μM) oxidation by H_2O_2 in PBS at pH 7.4 & 37 °C. (B) UV-Vis spectra's showing the reaction progress; (C) Reaction of **2h** with 10 mM H_2O_2. (E) Reaction of **2h** with 20 mM H_2O_2. (F) Reaction of **2h** with 40 mM H_2O_2. (F) Second-order rate determination of **2h** and H_2O_2. Figure represents a representative trail and average of three trials in shown in Table S9.*

*Table S9. Reaction rate of thioether **2h** oxidation by hydrogen peroxide*

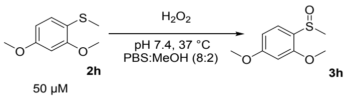

Trials	Pseudo-first order rate constant (s^{-1})			Second order rate constant (M^{-1} s^{-1})
	10 mM H_2O_2	20 mM H_2O_2	40 mM H_2O_2	
1	0.0000322	0.00007424	0.0001460	0.0038
2	0.00003898	0.00007757	0.00009616	0.0018
3	0.00005825	0.0001659	0.0002230	0.0051

1.12. Plot of second order reaction rate vs Hamett constant

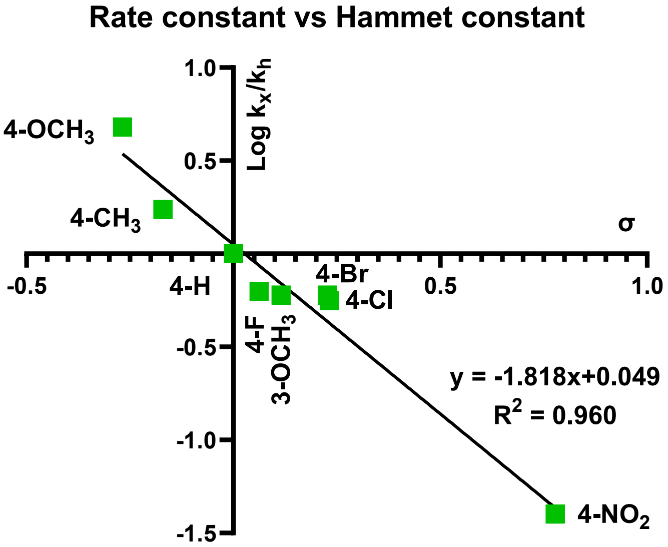

Figure S10. Effect of electronic substituent on the reaction rate of thioether oxidation by H_2O_2 based on the Hammett constant.

1.13. Second order rate determination of thioether 5 oxidation by H_2O_2

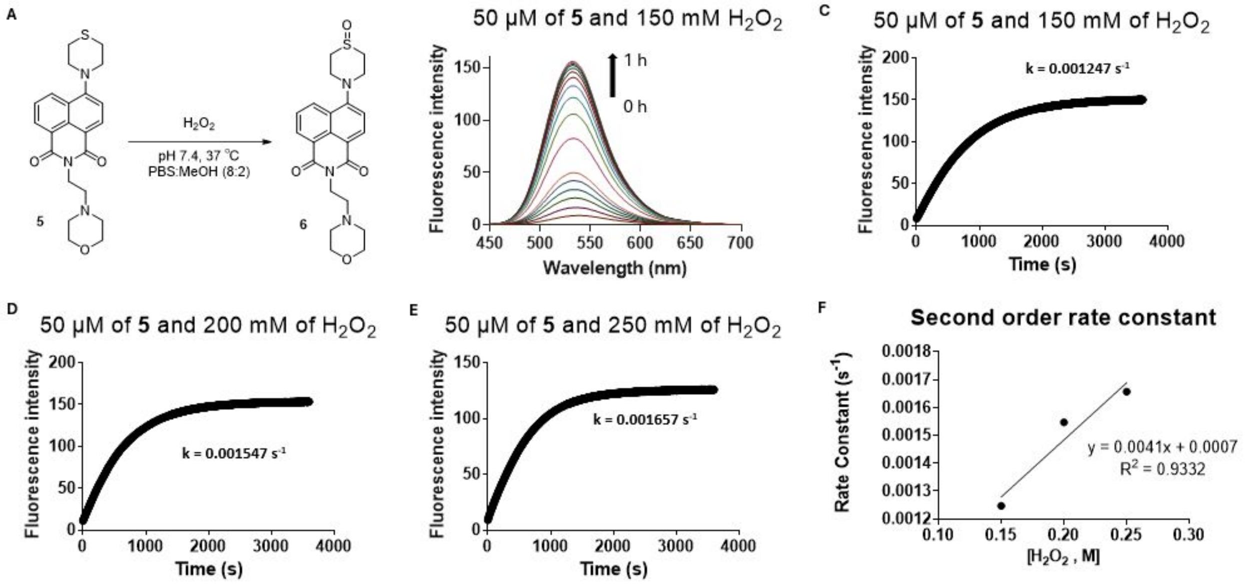

*Figure S11. (A) Reaction scheme of **5** (50 µM) oxidation by H_2O_2 in PBS at pH 7.4 & 37 °C. (B) UV-Vis spectral changes showing the reaction progression; (C) Reaction of **5** with 150 mM H_2O_2; (D) Reaction of **5** with 200 mM H_2O_2; (E) Reaction of **5** with 250 mM H_2O_2; (F) Second order rate determination of **5** and H_2O_2. Figure represents a representative trail and average of trials is shown in Table S10.*

*Table S10. Reaction rate of thioether **5** oxidation by hydrogen peroxide*

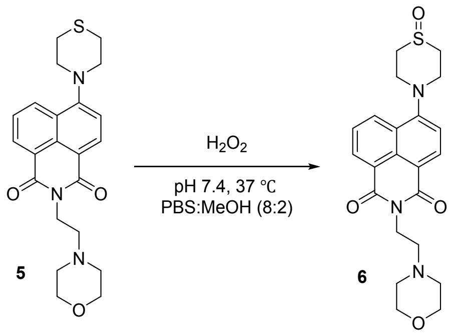

Trials	Pseudo-first order rate constant (s^{-1})			Second order rate constant (M^{-1} s^{-1})
	150 mM H_2O_2	200 mM H_2O_2	250 mM H_2O_2	
1	0.001247	0.001547	0.001657	0.0041
2	0.001277	0.001627	0.002014	0.0074
3	0.001290	0.001588	0.002150	0.0086

2. Oxidation of thioethers by NaOCl

2.1. Reaction of thioether 2a with NaOCl

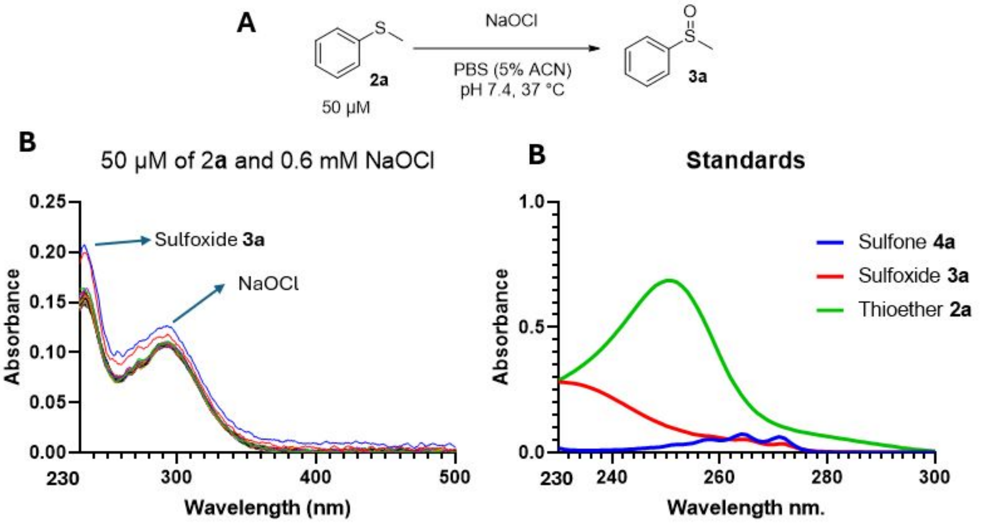

*Figure S12. (A) Reaction scheme of thioether **2a** oxidation by NaOCl in PBS (containing 5% ACN) at pH 7.4 and 37 °C. (B) UV-Vis spectra's showing the reaction progress of 50 μM thioether **2a** with 0.6 mM NaOCl at pH 7.4 and 37 °C; (C) Standard UV-Vis spectra's of **2a**, **3a**, and **4a**.*

2.2. Reaction of thioether 2a with NaOCl

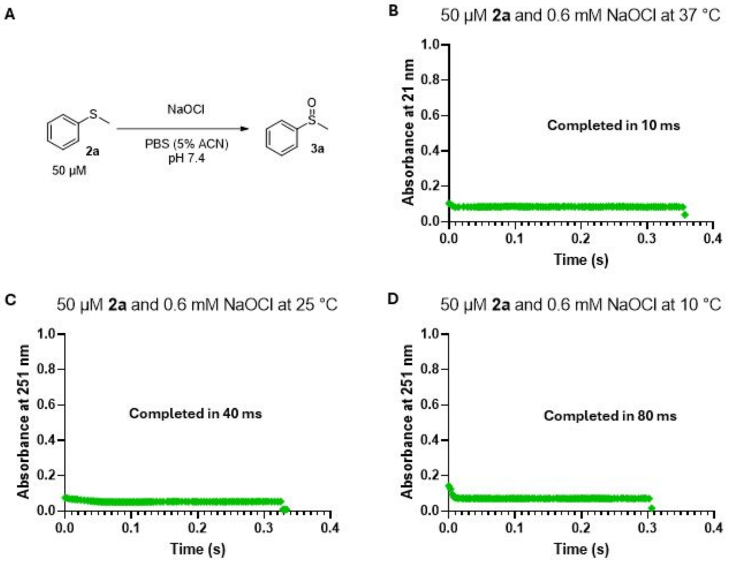

*Figure S13. (A) Reaction scheme of thioether **2a** oxidation by NaOCl in PBS (containing 5% ACN) at pH 7.4. (B) Time-resolved absorption spectra of 50 µM thioether **2a** with 0.6 mM NaOCl at 37 °C; (C) Time-resolved absorption spectra of 50 µM thioether **2a** with 0.6 mM NaOCl at 25 °C; (D) Time-resolved absorption spectra of 50 µM thioether **2a** with 0.6mM NaOCl at 10 °C.*

2.3. Second order rate determination of sulfoxide 3a oxidation by NaOCl

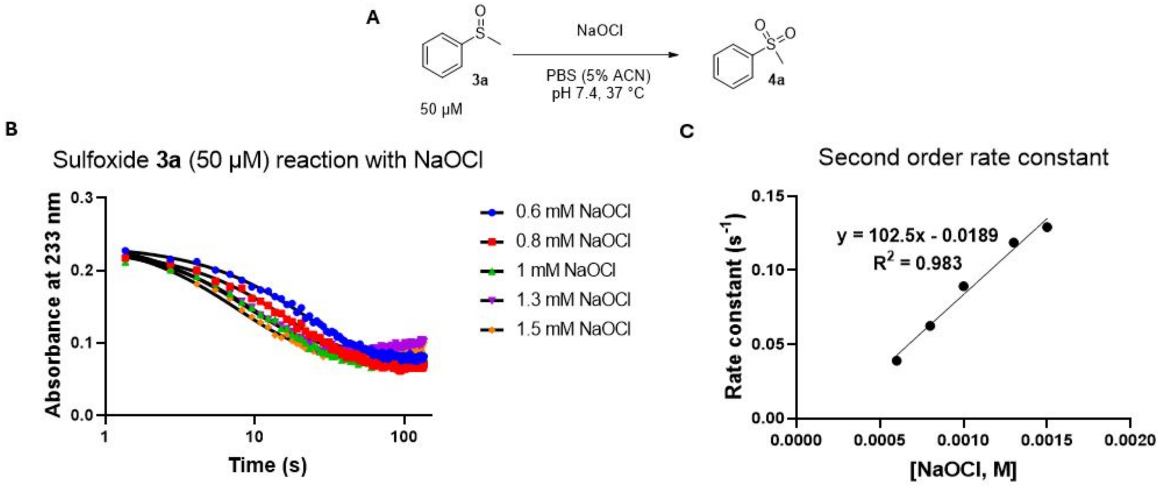

*Figure S14. (A) Sulfoxide **3a** oxidation by NaOCl in PBS (containing 5% ACN) at pH 7.4 and 37 °C; (B) Time-resolved absorption spectra of 50 µM sulfoxide **3a** with various concentrations of NaOCl (0.6 to 1.5 mM); and (C) Second-order rate determination of the reaction between sulfoxide **3a** and NaOCl.*

2.4.Reaction of thioether 2c-e with NaOCl

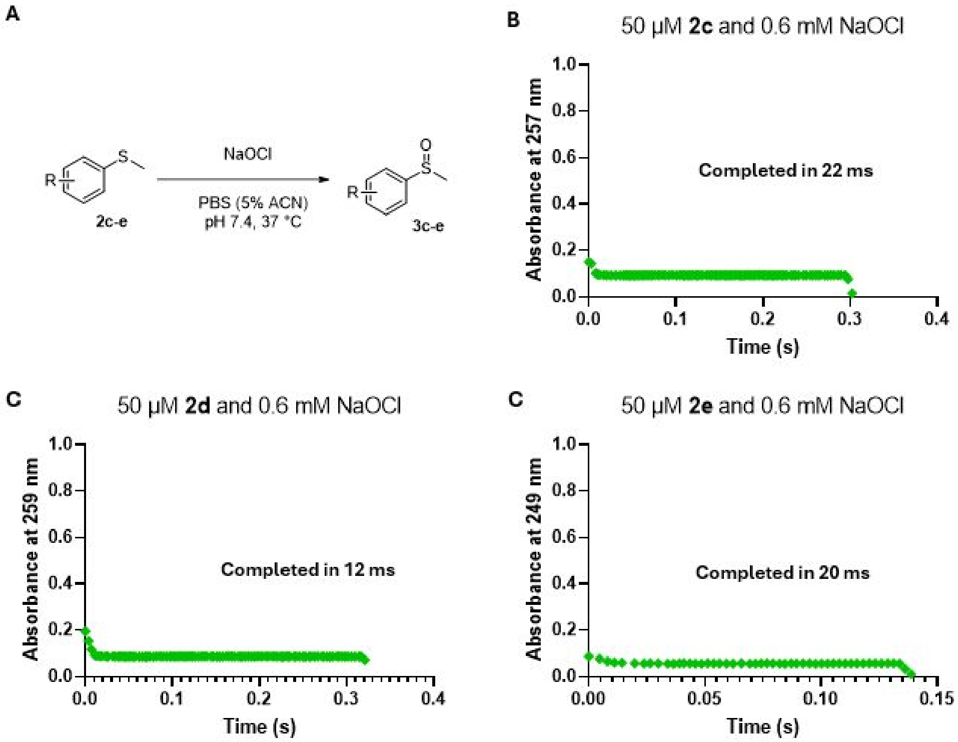

*Figure S15. (A) Reaction scheme of thioether oxidation by NaOCl in PBS (containing 5% ACN) at pH 7.4 and 37 °C. (B) Time-resolved absorption spectra of 50 μM **2c** with 0.6 mM NaOCl; (C) Time-resolved absorption spectra of 50 μM **2d** with 0.6 mM NaOCl; and (D) Time-resolved absorption spectra of 50 μM **2e** with 0.6 mM NaOCl.*

2.5. Second order rate determination of sulfoxide 3c oxidation by NaOCl

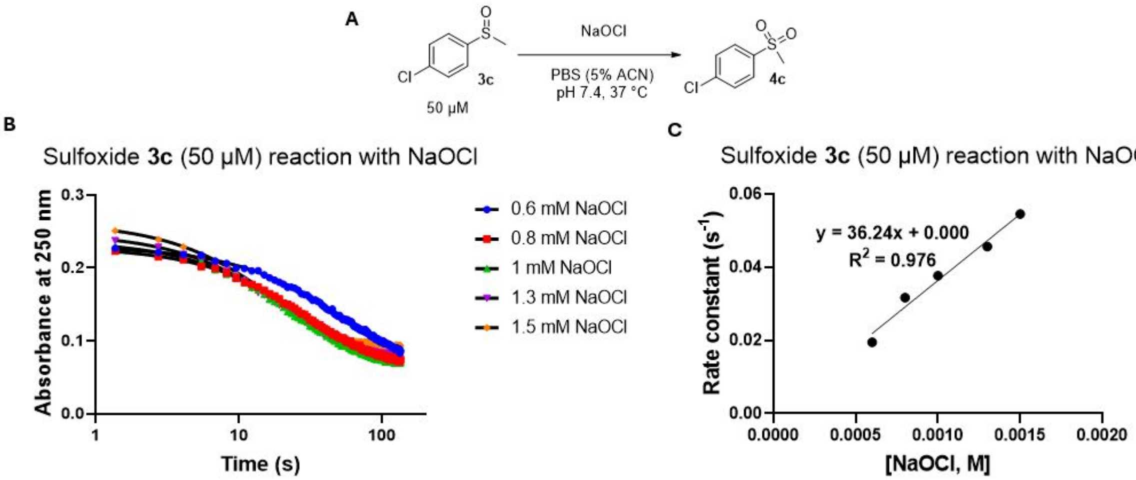

*Figure S16. (A) Sulfoxide **3c** oxidation by NaOCl in PBS (containing 5% ACN) at pH 7.4 and 37 °C; (B) Time-resolved absorption spectra of 50 μM sulfoxide **3c** with various concentrations of NaOCl (0.6 to 1.5 mM); and (C) Second-order rate determination of the reaction between sulfoxide **3c** and NaOCl.*

2.6. Second order rate determination of sulfoxide 3d oxidation by NaOCl

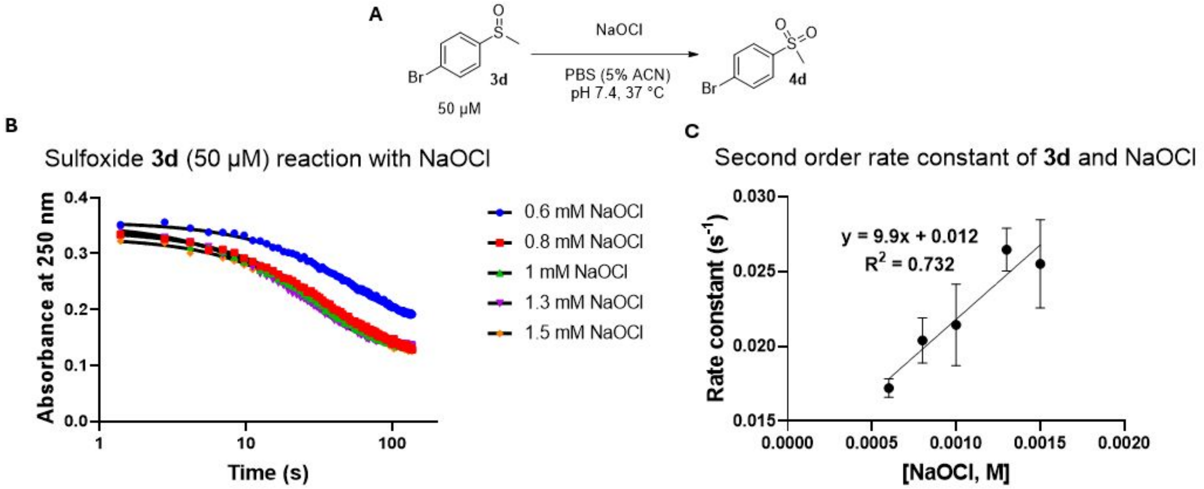

*Figure S17. (A) Sulfoxide **3d** oxidation by NaOCl in PBS (containing 5% ACN) at pH 7.4 and 37 °C; (B) Time-resolved absorption spectra of 50 μM sulfoxide **3d** with various concentrations of NaOCl (0.6 to 1.5 mM); and (C) Second-order rate determination of the reaction between sulfoxide **3d** and NaOCl.*

2.7. Second order rate determination of sulfoxide 3e oxidation by NaOCl

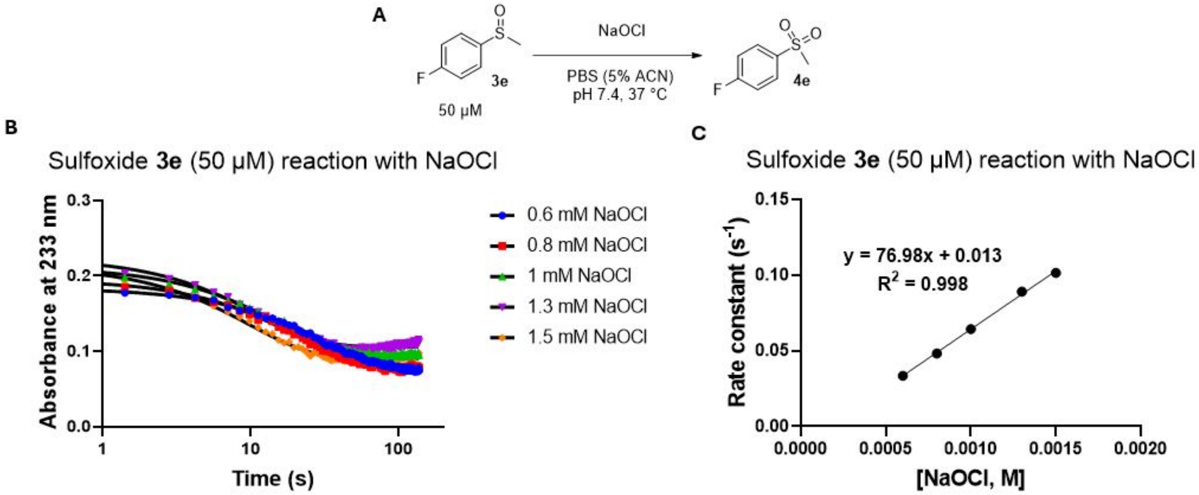

*Figure S18. (A) Sulfoxide **3e** oxidation by NaOCl in PBS (containing 5% ACN) at pH 7.4 and 37 °C; (B) Time-resolved absorption spectra of 50 μM sulfoxide **3e** with various concentrations of NaOCl (0.6 to 1.5 mM); and (C) Second-order rate determination of the reaction between sulfoxide **3e** and NaOCl.*

2.8. Second order rate determination of thioether 2b oxidation by NaOCl

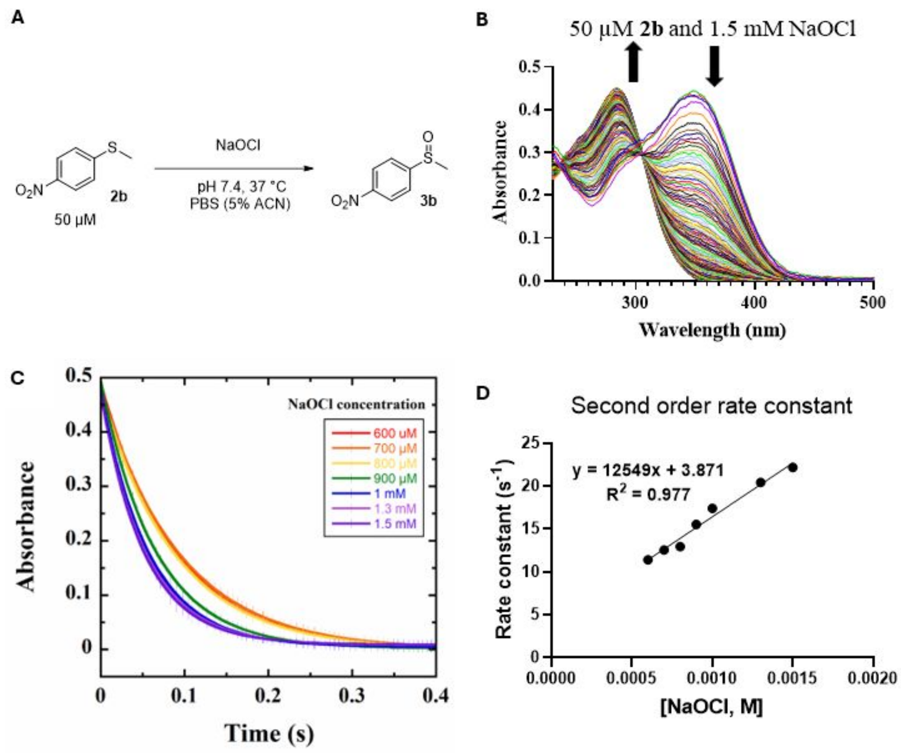

*Figure S19. (A) Thioether **2b** oxidation by NaOCl in PBS (containing 5% ACN) at pH 7.4 at 37 °C. (B) UV-Vis spectra showing the reaction progress. (C) Time-resolved absorption spectra of 50 µM thioether **2b** with various concentrations of NaOCl (0.6 to 1.5 mM). (D) Second-order rate determination of thioether **2b** and NaOCl.*

2.9. Second order rate determination of sulfoxide 3b oxidation by NaOCl

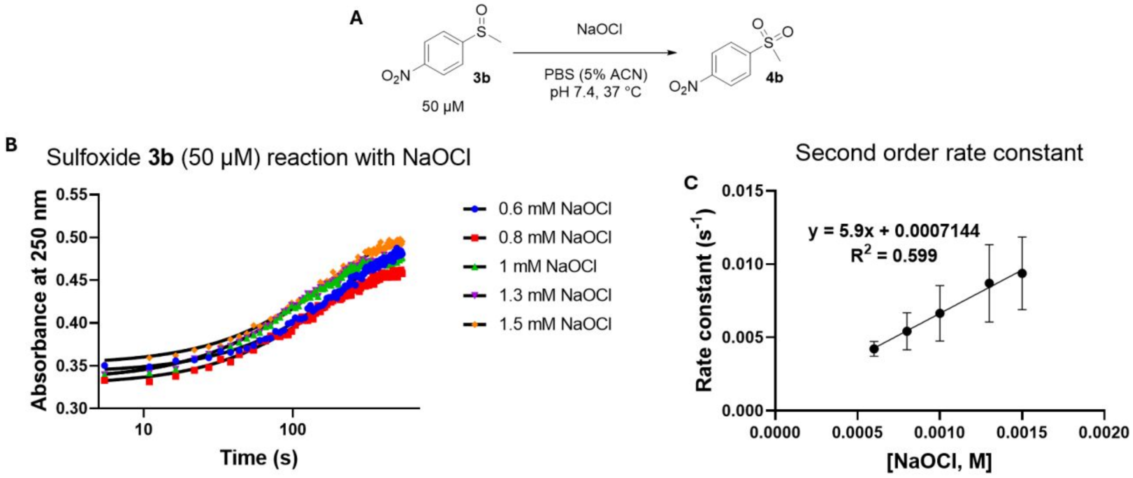

*Figure S20. (a) Sulfoxide **3b** oxidation by NaOCl in PBS (containing 5% ACN) at pH 7.4 at 37 °C. (b) Time-resolved absorption spectra of 50 µM sulfoxide **3b** with various concentrations of NaOCl (0.6 to 1.5 mM). (c) Second-order rate determination of sulfoxide **3b** and NaOCl.*

2.10. A proposed reaction mechanism for the NaOCl mediated oxidation of thioether to sulfoxide and to sulfone

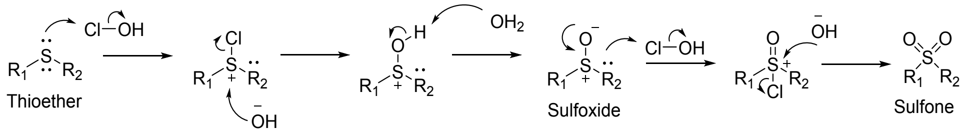

Scheme S2. A proposed reaction mechanism for the NaOCl mediated oxidation of thioether to sulfoxide and to sulfone.

2.11. Plot of second order reaction rate vs Hamett constant

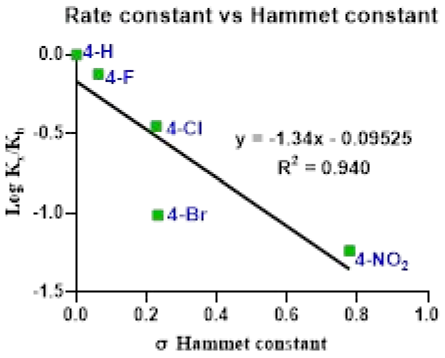

Figure S21. Effect of substituent on the reaction rate of sulfoxide oxidation by NaOCl based on the Hammett constant.

2.12. Reaction rate determination of thioether 5 oxidation by NaOCl

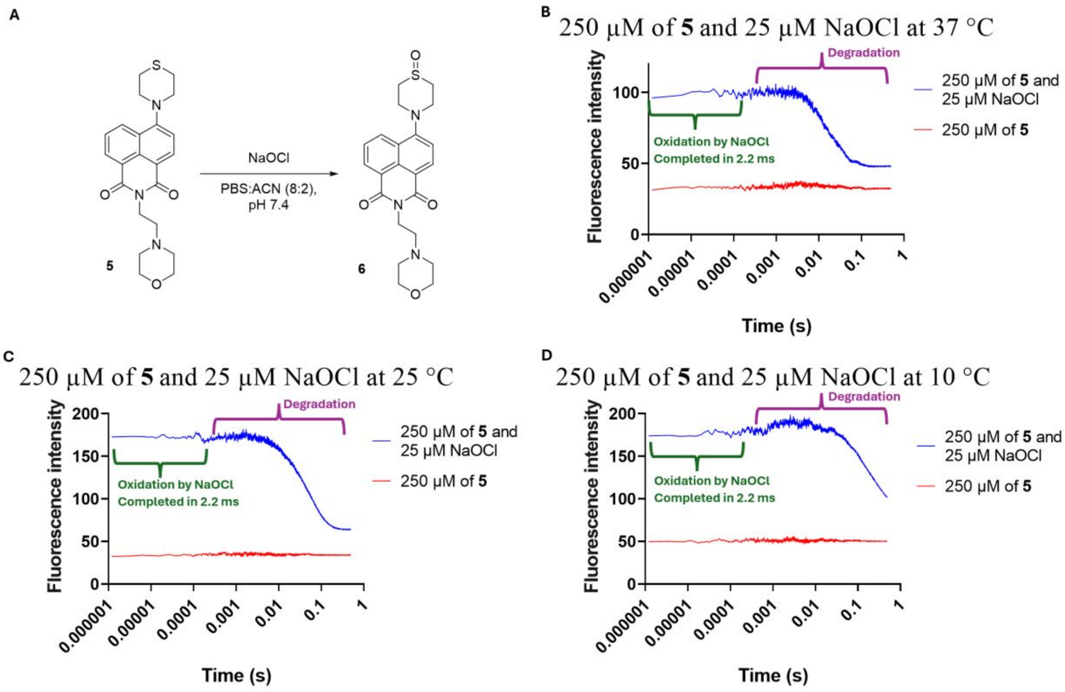

*Figure S22. (A) Thioether **5** oxidation by NaOCl in PBS (containing 5% ACN) at pH 7.4. (B) Time-resolved absorption spectra of 250 μM of **5** with 25 μM NaOCl at 37 °C; (C) Time-resolved absorption spectra of 250 μM of **5** with 25 μM NaOCl at 25 °C; and (D) Time-resolved absorption spectra of 250 μM of **5** with 25 μM NaOCl at 10 °C.*

3. UV-Vis spectra and molar extinction coefficients (ε) of thioethers, sulfoxide, and sulfone

3.1. UV-Vis spectra as standards

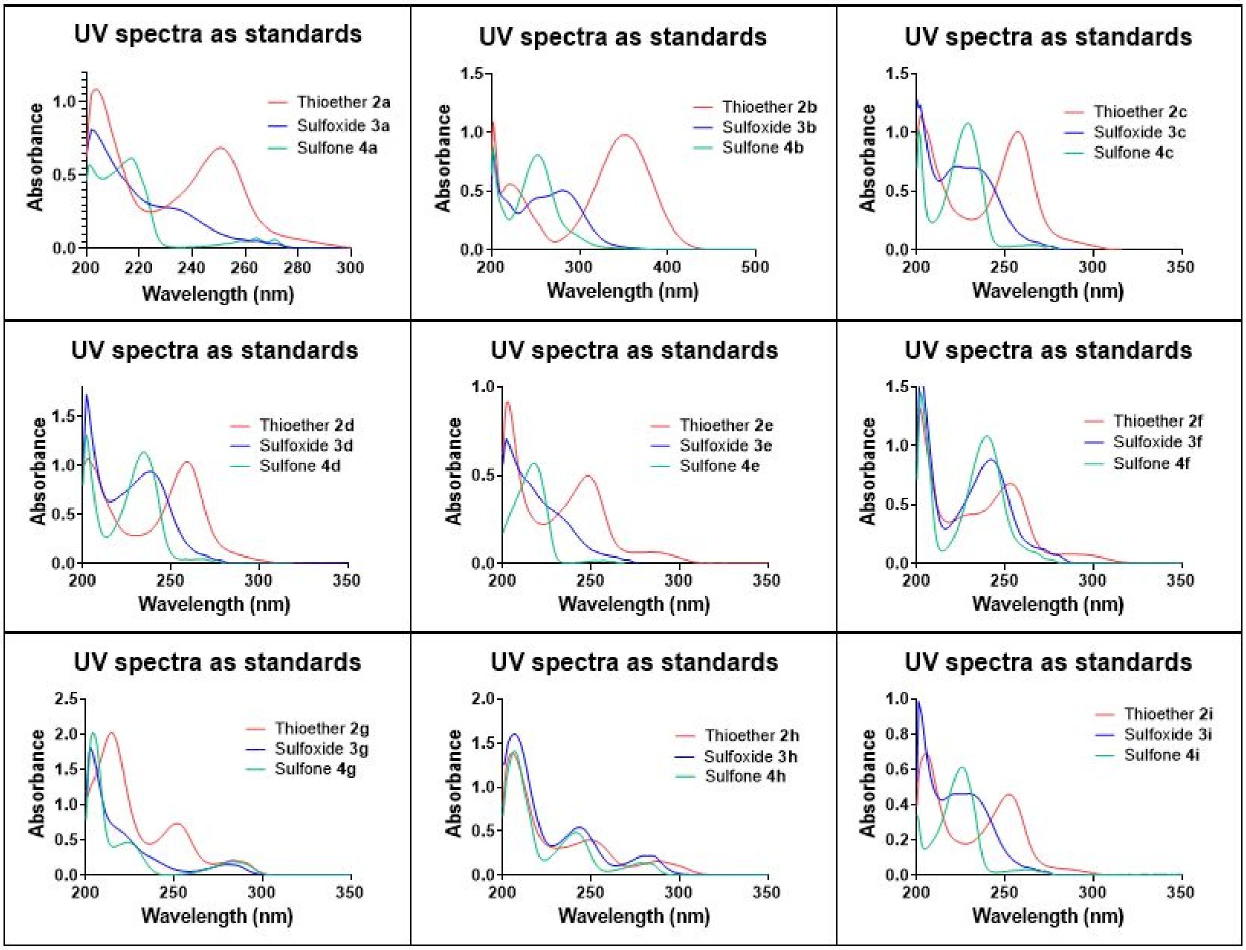

Figure S23. UV-Vis spectrums of thioethers, sulfoxide, and sulfone. Molar extinction coefficients (ε) of thioethers, sulfoxide, and sulfone has been summarized in table S11.

3.2. Molar extinction coefficients (ε)

Table S11. Molar extinction coefficients (ε) of thioethers, sulfoxide, and sulfone

Thioether			Sulfoxide			Sulfone		
#	λ_{max} (nm)	ε ($M^{-1}cm^{-1}$)	#	λ_{max} (nm)	ε ($M^{-1}cm^{-1}$)	#	λ_{max} (nm)	ε ($M^{-1}cm^{-1}$)
**2a**	251	13,740	**3a**	202	16,240	**4a**	216	12,180
**2b**	353	19,580	**3b**	280	10,120	**4b**	252	16,200
**2c**	257	20,160	**3c**	222	14,260	**4c**	229	21,660
**2d**	259	20,800	**3d**	238	18,740	**4d**	235	22,760
**2e**	248	9,980	**3e**	202	14,140	**4e**	218	11,380
**2f**	251	13,400	**3f**	242	17,680	**4f**	239	21,640
**2g**	215	40,500	**3g**	203	36,100	**4g**	204	40,560

**2h**	205	27,840	**3h**	207	32,060	**4h**	206	28,220
**2i**	205	13,820	**3i**	201	19,780	**4i**	226	12,280

4. Experimental section

4.1. Materials and Methods

Chemical reagents were purchased from the following: Sigma-Aldrich (Saint Louis, Missouri, USA), Ambeed (Illinois, USA), Combi-Blocks (California, USA), and Oakwood (Estill, South Carolina, USA). Compounds **2a-i**; **3a, c, d, e, g,** and **i**; and **4c-g,** and **i** were purchased commercially and used directly without further purification. ^{1}H NMR (400 MHz) spectra and ^{13}C-NMR (100 MHz) were recorded on a Bruker Avance 400 MHz NMR spectrometer in deuterated solvent from Oakwood Products, Inc. (South Carolina, USA). Chemical shifts were reported as δ values (ppm). TMS (δ = 0.00 ppm) or residual peaks of the deuterated solvent were used as the internal reference. NaOCl solution (CAS 7681-52-9) was purchased from Sigma-Aldrich (Massachusetts, USA). The concentration of NaOCl solution was determined to be 1.811 M using UV-Vis spectrophotometer at 292 nm (ε: 360 $M^{-1}cm^{-1}$). UV-Vis absorption spectra were obtained using a Shimadzu PharmaSpec UV-1700 UV-Visible spectrophotometer (Kyoto, Japan). Mass spectrometric analyses were conducted by the Georgia State University Mass Spectrometry Facilities. The milligram scale quantities were weighed on a C-33 microbalance (CAHN Instruments Inc., California, USA). For the stopped-flow, SF-61DX2 Hi-Tech KinetAsyst performance stopped-flow spectrophotometer was used. UV-Vis spectra of thioether, sulfoxide, and sulfone were recorded and were used as reference (Figure S23).

4.2. Reaction rate determination of thioether oxidation by H_2O_2:

First, the 10 mM stock solution of compounds were prepared in methanol. Then, 50 μM of thioether analog was incubated with different concentrations of H_2O_2 (5-40 mM) in PBS (containing 20% MeOH) at pH 7.4. The resulting reaction mixture was incubated at 37 °C. UV-spectra was recorded at pre-designated time points. For the data analysis, time resolved data was fitted into a one-phase exponential equation to obtain pseudo first-order rate constant. Then, the pseudo first-order rate constant was plotted against H_2O_2 concentration, and the second-order rate constant was calculated from the slope.

Equation for one-phase exponential curve: -A*exp(-k*X) + C

Where k represents the reaction rates, A represents the absorbance change, and C represents the final absorbance.

The following protocols were used for the reaction of thioethers with different concentrations of H_2O_2 (5-40 mM):

50 μM of thioether and 5 mM H_2O_2: Firstly, 15 μL of 10 mM thioether was added to 585 μL of methanol followed by addition of 2385 μL of PBS at pH 7.4. Then, 15 μL of 1 M H_2O_2 was added to the solution and resulting reaction mixture was incubated at 37 °C. UV-spectra was recorded at pre-designated time points.

50 μM of thioether and 10 mM H_2O_2: Firstly, 15 μL of 10 mM of thioether was added to 585 μL of methanol followed by the addition of 2370 μL of PBS at pH 7.4. Then, 30 μL of 1 M H_2O_2 was added to the solution and resulting reaction mixture was incubated at 37 °C. UV-spectra was recorded at pre-designated time points.

50 μM of thioether and 20 mM H_2O_2: Firstly, 15 μL of 10 mM stock of thioether was added to 585 μL of methanol followed by the addition of 2340 μL of PBS at pH 7.4. Then, 60 μL of 1 M H_2O_2 was added to the solution and resulting reaction mixture was incubated at 37 °C. UV-spectra was recorded at pre-designated time points.

50 μM of thioether and 40 mM H_2O_2: First, 15 μL of 10 mM thioether was added to 585 μL of methanol followed by the addition of 2280 μL of PBS at pH 7.4. Then, 120 μL of 1 M H_2O_2 was added to the solution and resulting reaction mixture was incubated at 37 °C. UV-spectra was recorded at pre-designated time points.

4.3. Reaction rate determination of aliphatic thioether (5) oxidation by H_2O_2:

First, - 10 mM stock solution of compound was prepared in DMF. Then, 50 μM of thioether was incubated with different concentrations of H_2O_2 (150-250 mM) in PBS (containing 20% MeOH) at pH 7.4. The resulting reaction mixture was incubated at 37 °C. Fluorescence-spectra were recorded at pre-designated time points (λ_{ex} 410 nm and λ_{em} 530 nm). For the data analysis, time-resolved data were fitted into a one-phase exponential equation to obtain pseudo first-order rate constant. Then, the pseudo first-order rate constant was plotted against H_2O_2 concentration, and the second-order rate constant was calculated from the slope.

4.4. Reaction rate determination of thioether/sulfoxide oxidation by NaOCl:

First, the 10 mM stock solution of compounds were prepared in acetonitrile. Working solutions were prepared in PBS at 2× the final reaction solution as they get mixed in a one-to-one ratio in stopped-flow. Working solution of 100 μM of thioether/sulfoxide was prepared by adding 0.5 mL of 10 mM thioether/sulfoxide to 49.5 mL PBS (containing 4.5 mL acetonitrile) at pH 7.4. Working solutions of NaOCl (1.2- to 3- mM) was prepared in PBS at pH 7.4. Solutions were loaded into syringes and mounted into the stopped-flow spectrophotometer. The solutions were mixed in a one-to-one ratio in a single mixing mode with an instrument mixing time of 2.2 ms, and the spectral changes were then recorded at 37 °C. For

thioanisole analog, the reaction was carried out using a similar protocol at two additional temperatures 10 °C and 25 °C. For the data analysis, time resolved data was fitted into a one-phase exponential equation to obtain pseudo first-order rate constant. Then, the pseudo first-order rate constant was plotted against NaOCl concentration, and the second-order rate constant was calculated from the slope.

4.5. Reaction rate determination of aliphatic thioether (5) oxidation by NaOCl:

First, a-10 mM stock solution of compound **5** was prepared in DMF. Working solutions were prepared in PBS at 2× the final reaction solution as they get mixed in a one-to-one ratio in stopped-flow. Working solution of 500 μM of thioether was prepared by adding 0.75 mL of 10 mM thioether to 14.25 mL PBS (containing 20% acetonitrile) at pH 7.4. Working solution of NaOCl (50 μM) was prepared in PBS at pH 7.4. Solutions were loaded into syringes and mounted into the stopped-flow spectrophotometer. The solutions were mixed in a one-to-one ratio in a single mixing mode with an instrument mixing time of 2.2 ms, and the spectral changes were then recorded at 37 °C, 25 °C and 10 °C. Time resolved fluorescent change was monitored at λ_{ex} 410 nm and λ_{em} 530 nm.

4.6. Chemistry

(2,4-Dimethoxyphenyl)(methyl)sulfane (2h): To a solution of 2,4-dimethoxybenzenethiol (40 mg, 33.7 μL, 0.23 mmol) in dichloromethane (5 mL), iodomethane (50 mg, 21.9 μL, 0.35 mmol), and triethylamine (59 mg, 81.9 μL, 0.59 mmol) were added. The reaction mixture was allowed to stir at room temperature for 3 h. The TLC showed the completion of the reaction. The reaction was then quenched with water. The product was extracted using 3×30 mL DCM. The organic layers were combined and dried over anhydrous Na_2SO_4. The DCM layer was filtered, and the filtrate was evaporated under vacuum. The crude was purified by silica get column using hexane/ethyl acetate (2:1) to obtain the product as a yellowish white liquid. Isolated yield 38 mg, 89%. ^{1}H NMR ($CDCl_3$) δ 7.22 (d, *J* = 8.0 Hz, 1H), 6.51 (d, *J* = 8.0 Hz, 1H), 6.49 (s, 1H), 3.89 (s, 3H), 3.82 (s, 3H), 2.40 (s, 3H). ^{13}C NMR ($CDCl_3$) δ 159.8, 158.5, 130.4, 117.0, 104.8, 98.9, 55.8, 55.5, 16.6. HRMS calculated for $C_9H_{13}O_2S$ $(M+H)^+$: 185.0636; found: 185.0626.

To synthesize corresponding sulfoxide and sulfones the following procedure was used: To a RBF containing DCM, thioether (1 eq.) and 3-chlorobenzoperoxoic acid (mCPBA) (0.9 eq to synthesize sulfoxide and 3 eq. to synthesize sulfone) were added. The reaction mixture was allowed to stir at room temperature. Completion of the reaction was monitored by TLC. The reaction was then quenched with water and $NaHCO_3$ solution. The product was extracted using 3×30 mL DCM. The organic layers were combined and dried over anhydrous Na_2SO_4. The DCM layer was filtered, and the filtrate was evaporated under vacuum. The crude was purified by silica get column to obtain pure product.

1-(Methylsulfinyl)-4-nitrobenzene (3b): White crystalline solid. Isolated yield 95 mg, 97%. ^{1}H NMR ($CDCl_3$) δ 8.41 (d, *J* = 8.8 Hz, 2H), 7.85 (d, *J* = 8.8 Hz, 2H), 2.81 (s, 3H); ^{13}C NMR ($CDCl_3$) δ 153.2, 149.5, 124.7, 124.5, 43.9. HRMS calculated for $C_7H_8NO_3S$ $(M+H)^+$: 186.0225; found: 186.0229. Data matches with the literature reports.[1]

1-Methoxy-4-(methylsulfinyl)benzene (3f): Whitish pale yellow crystalline solid. Isolated yield 123 mg, 99%. ^{1}H NMR ($CDCl_3$) δ 7.60 (d, *J* = 8.8 Hz, 2H), 7.04 (d, *J* = 8.8 Hz, 2H), 3.86 (s, 3H), 2.70 (s, 3H);^{13}C NMR ($CDCl_3$) δ 161.9, 136.6, 125.4, 114.8, 55.5, 44.0; HRMS calculated for $C_8H_{11}O_2S$ $(M+H)^+$: 171.0480; found: 171.0484. Data matches with the literature reports.[1]

2,4-dimethoxy-1-(methylsulfinyl)benzene (3h): Pale yellow liquid. Isolated yield 48 mg, 45%. ^{1}H NMR ($CDCl_3$) δ 7.63 (d, *J* = 8.5 Hz, 1H), 6.61 (d, *J* = 8.6 Hz, 1H), 6.40 (s, 1H), 3.78 (s, 3H), 3.77 (s, 3H), 2.67 (s, 3H). ^{13}C NMR ($CDCl_3$) δ 163.7, 156.4, 126.3, 124.7, 105.7, 99.1, 56.1, 56.0, 41.9. HRMS calculated for $C_9H_{13}O_3S$ $(M+H)^+$: 201.0585; found: 201.0588.

(Methylsulfonyl)benzene (4a): White crystalline solid. Isolated yield 148 mg, 94%.^{1}H NMR ($CDCl_3$) δ 7.98 (d, *J* = 7.1 Hz, 2H), 7.69 (t, *J* = 7.4 Hz, 1H), 7.60 (t, *J* = 7.5 Hz, 2H), 3.08 (s, 3H). ^{13}C NMR ($CDCl_3$) δ 140.5, 133.7, 129.4, 127.3, 44.5. HRMS calculated for $C_7H_9O_2S$ $(M+H)^+$: 157.0323; found: 157.0314. Data matches with the literature reports.[2]

1-(Methylsulfonyl)-4-nitrobenzene (4b): White to pale yellow solid. Isolated yield 192 mg, 95%. ^{1}H NMR ($CDCl_3$) δ 8.44 (d, *J* = 8.8 Hz, 2H), 8.17 (d, *J* = 8.8 Hz, 2H), 3.13 (s, 3H). ^{13}C NMR ($CDCl_3$) δ 150.8, 145.9, 129.0, 124.6, 44.3. HRMS calculated for $C_7H_8NO_4S$ $(M+H)^+$: 202.0174; found: 202.0164. Data matches with the literature reports.[3]

2,4-dimethoxy-1-(methylsulfonyl)benzene (4h): White crystalline solid. Isolated yield 103 mg, 89%. ^{1}H NMR ($CDCl_3$) δ 7.78 (d, J = 8.7 Hz, 1H), 6.49 (d, J = 8.7 Hz, 1H), 6.46 (s, 1H), 3.88 (s, 3H), 3.79 (s, 3H), 3.09 (s, 3H). ^{13}C NMR ($CDCl_3$) δ 165.9, 159.0, 131.7, 120.9, 104.9, 99.7, 56.9, 56.1, 43.4. HRMS calculated for $C_9H_{13}O_4S$ $(M+H)^+$: 217.0535; found: 217.0543.

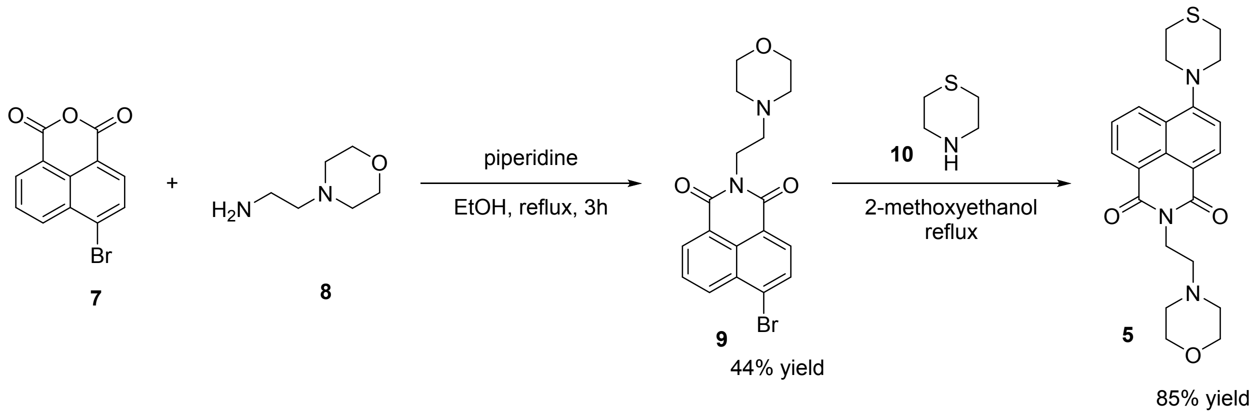

*Scheme S3. Synthesis scheme of probe **5**.*

6-bromo-2-(2-morpholinoethyl)-1H-benzo[de]isoquinoline-1,3(2H)-dione (9): Compound was synthesized based on the reported procedure.[4] Light yellow solid. Isolated yield 660 mg, 85%. ^{1}H NMR ($CDCl_3$) δ 8.60 (d, *J* = 7.2 Hz, 1H), 8.51 (d, *J* = 8.4 Hz, 1H), 8.36 (d, *J* = 7.8 Hz, 1H), 8.00 (d, *J* = 7.8 Hz, 1H), 7.81 (t, *J* = 7.9 Hz, 1H), 4.32 (t, *J* = 6.8 Hz, 2H), 3.68 (s, 4H), 2.71 (t, *J* = 6.6 Hz, 2H), 2.61 (s, 4H). HRMS calculated for $C_{18}H_{18}N_2O_3Br$ $(M+H)^+$: 389.0501; found: 389.0486.

2-(2-morpholinoethyl)-6-thiomorpholino-1H-benzo[de]isoquinoline-1,3(2H)-dione (5): Compound was synthesized based on the reported procedure.[5] Yellow solid. Isolated yield 90 mg, 44%. ^{1}H NMR ($CDCl_3$) δ 8.58 (dd, *J* = 7.3, 0.9 Hz, 1H), 8.51 (d, *J* = 8.0 Hz, 1H), 8.37 (dd, *J* = 8.4, 0.9 Hz, 1H), 7.71 (dd, *J* = 8.3, 7.4 Hz, 1H), 7.24 (d, *J* = 8.1 Hz, 1H), 4.38 (s, 2H), 3.75 (s, 4H), 3.59 – 3.45 (m, 4H), 3.04 – 2.91 (m, 4H), 2.73 (d, *J* = 39.2 Hz, 6H). HRMS calculated for $C_{22}H_{26}N_3O_3S$ $(M+H)^+$: 412.1695; found: 412.1695.

NMR's

^{1}H NMR (400 MHz)

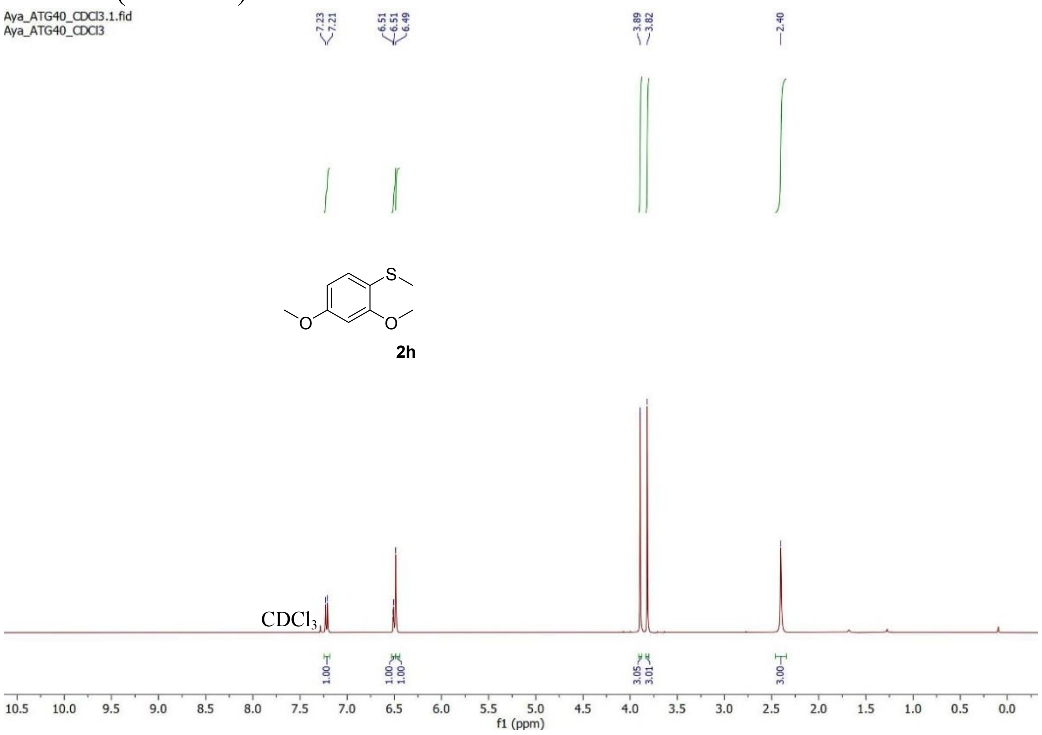

^{13}C-NMR (100 MHz)

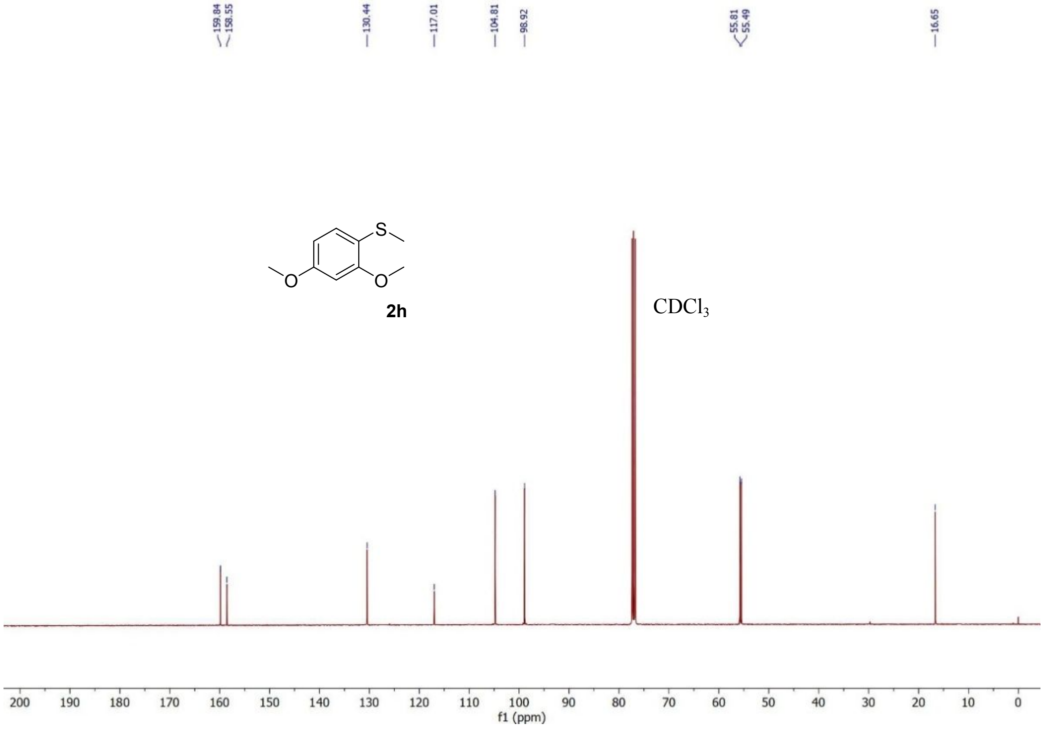

^{1}H NMR (400 MHz)

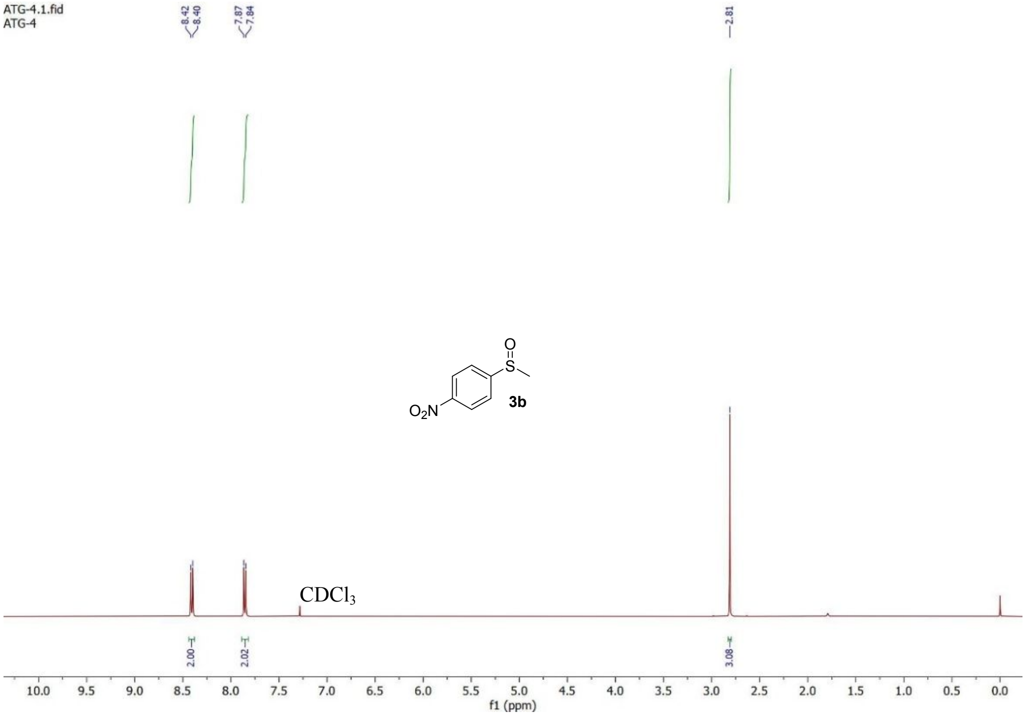

^{13}C-NMR (100 MHz)

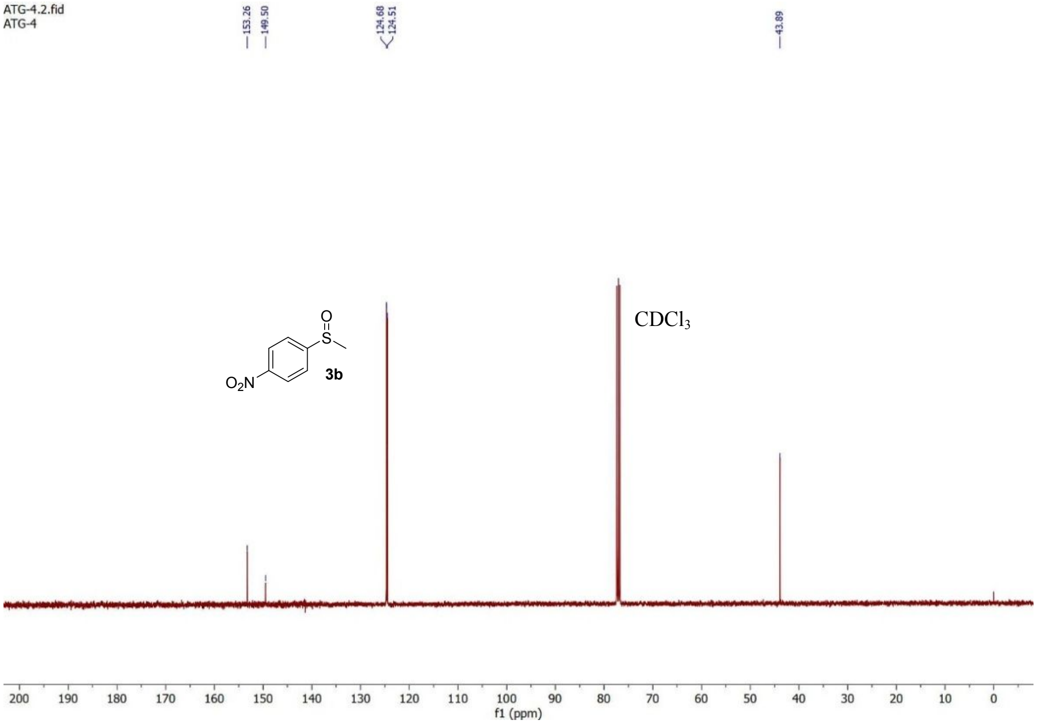

^{1}H NMR (400 MHz)

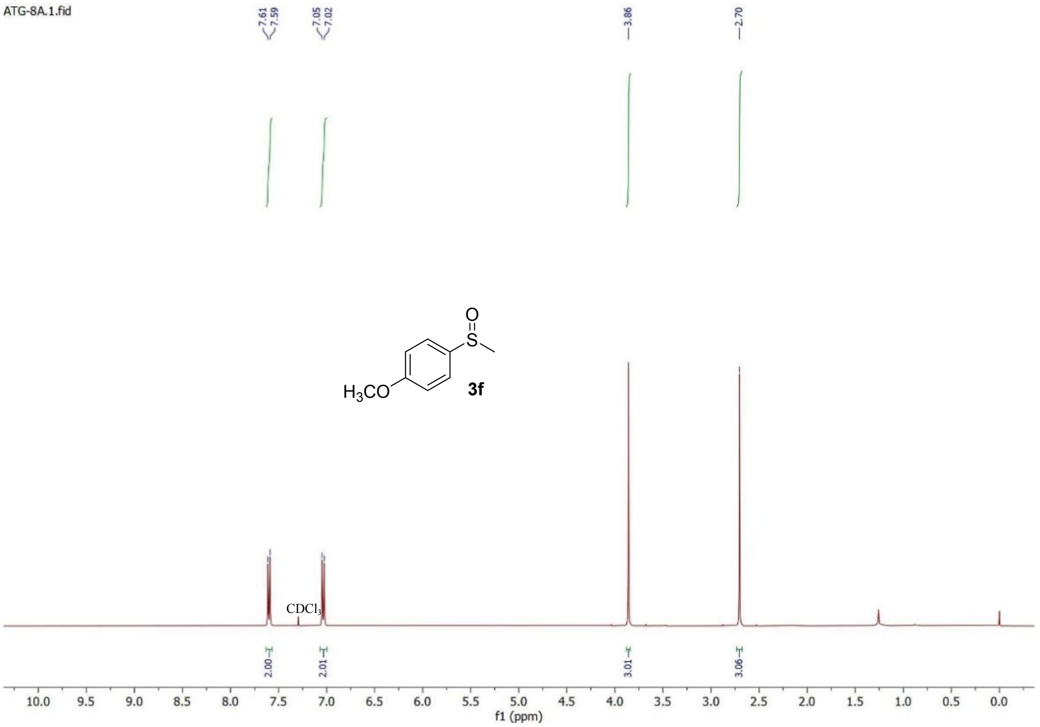

^{13}C-NMR (100 MHz)

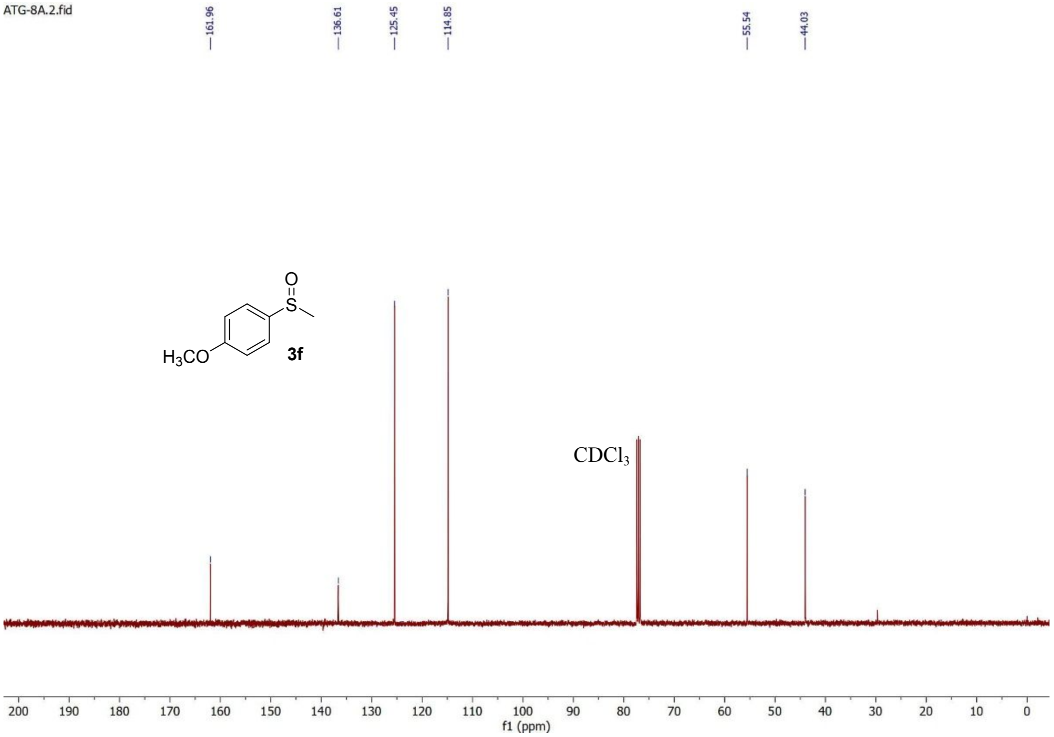

^{1}H NMR (400 MHz)

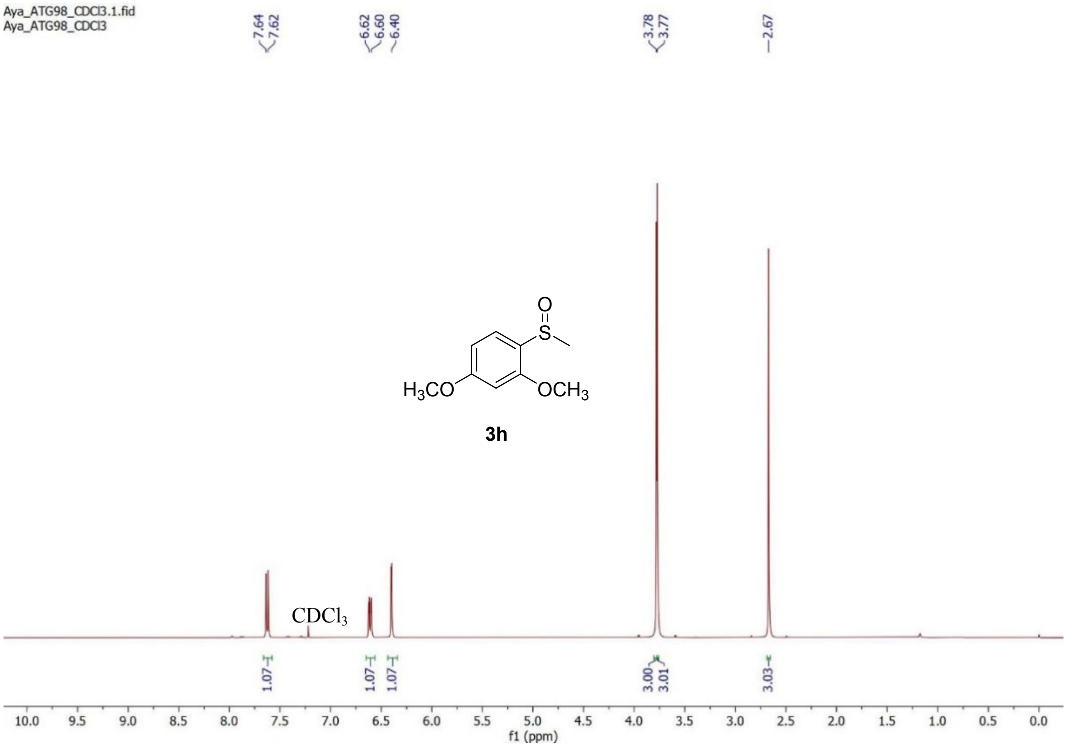

^{13}C-NMR (100 MHz)

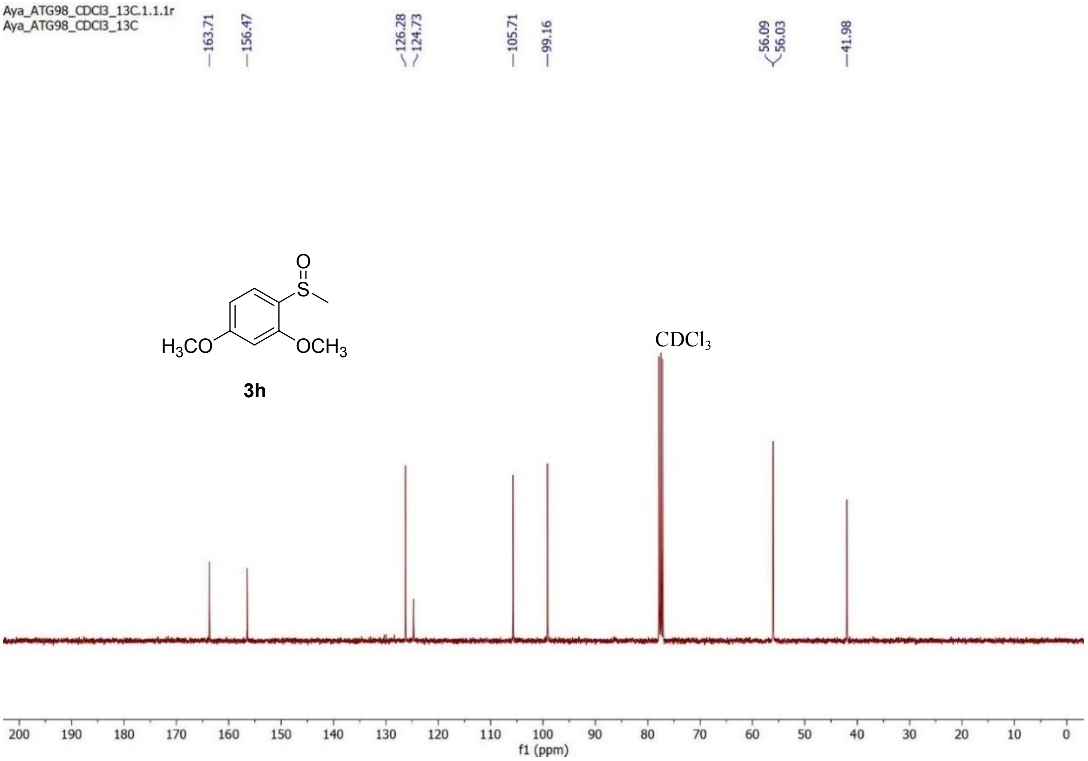

^{1}H NMR (400 MHz)

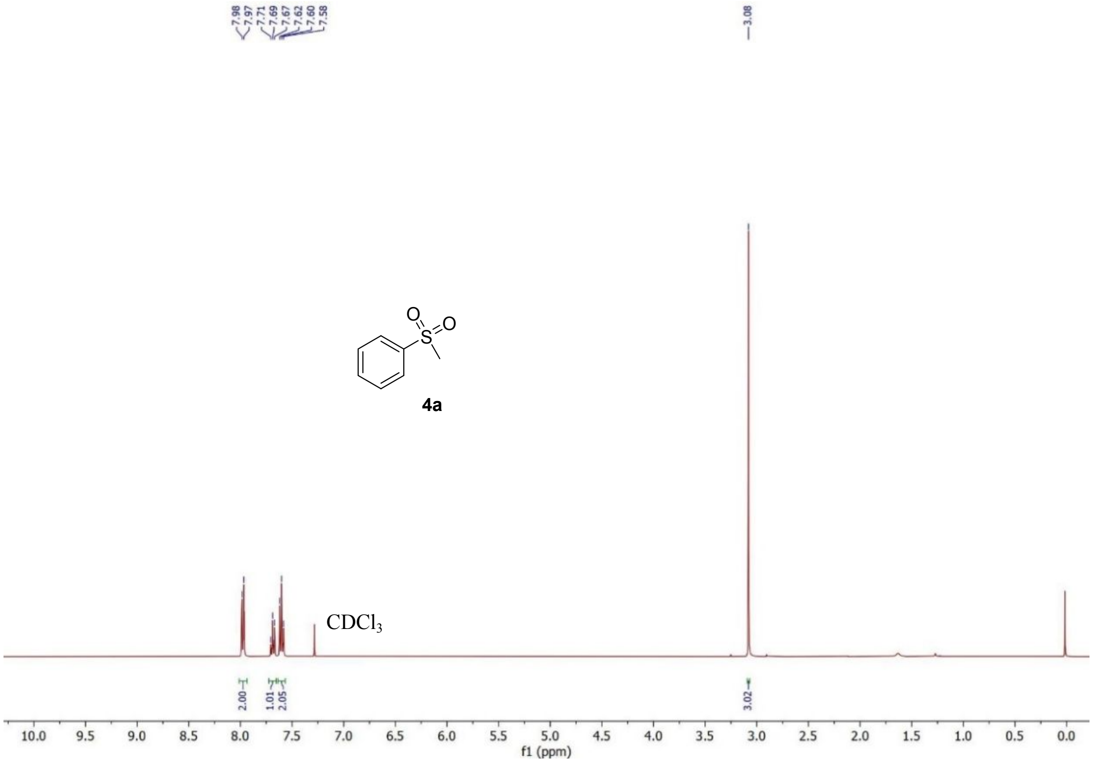

^{13}C-NMR (100 MHz)

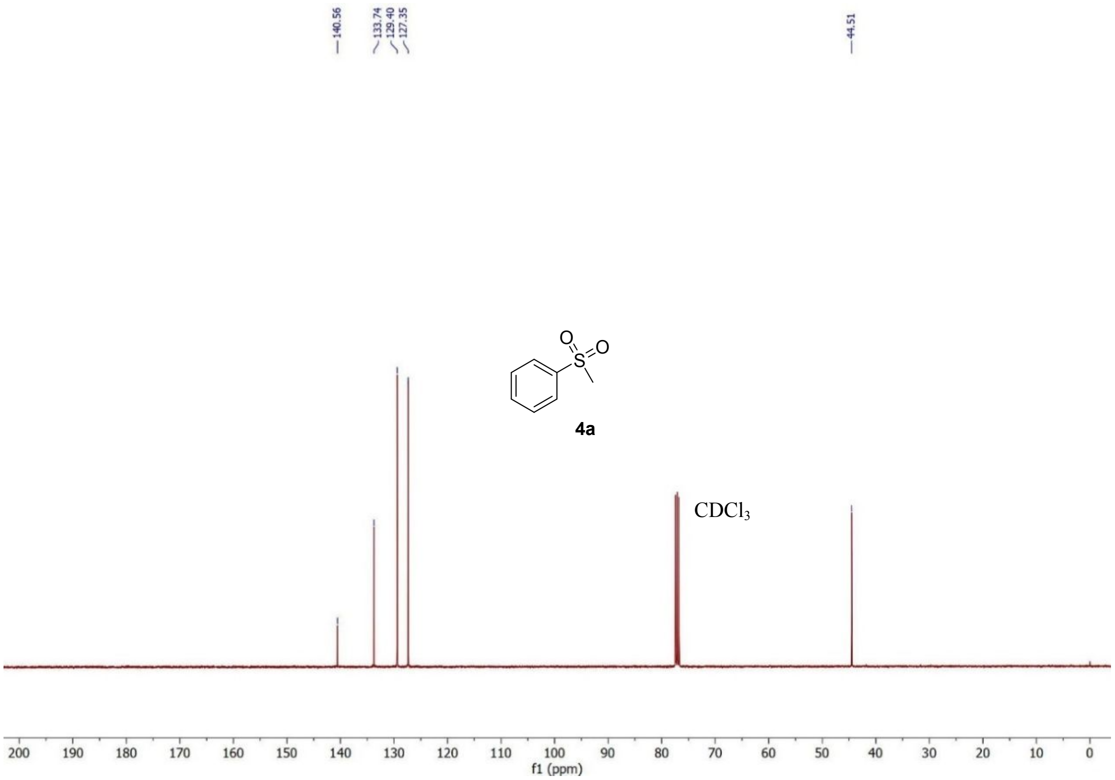

^{1}H NMR (400 MHz)

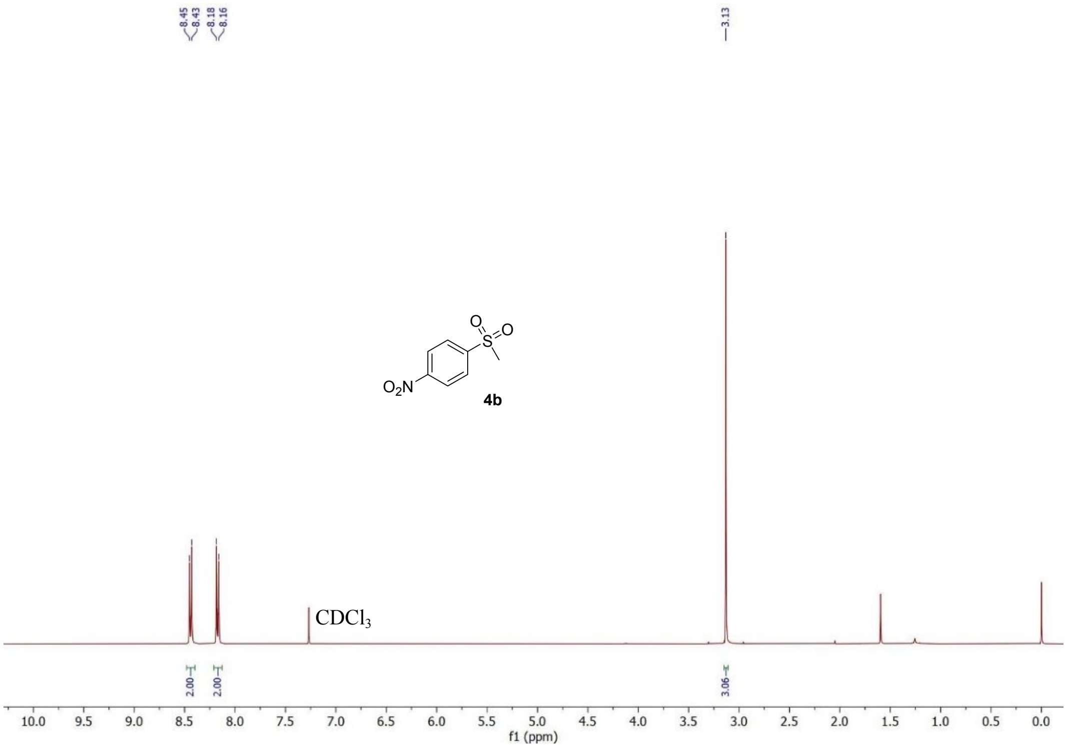

^{13}C-NMR (100 MHz)

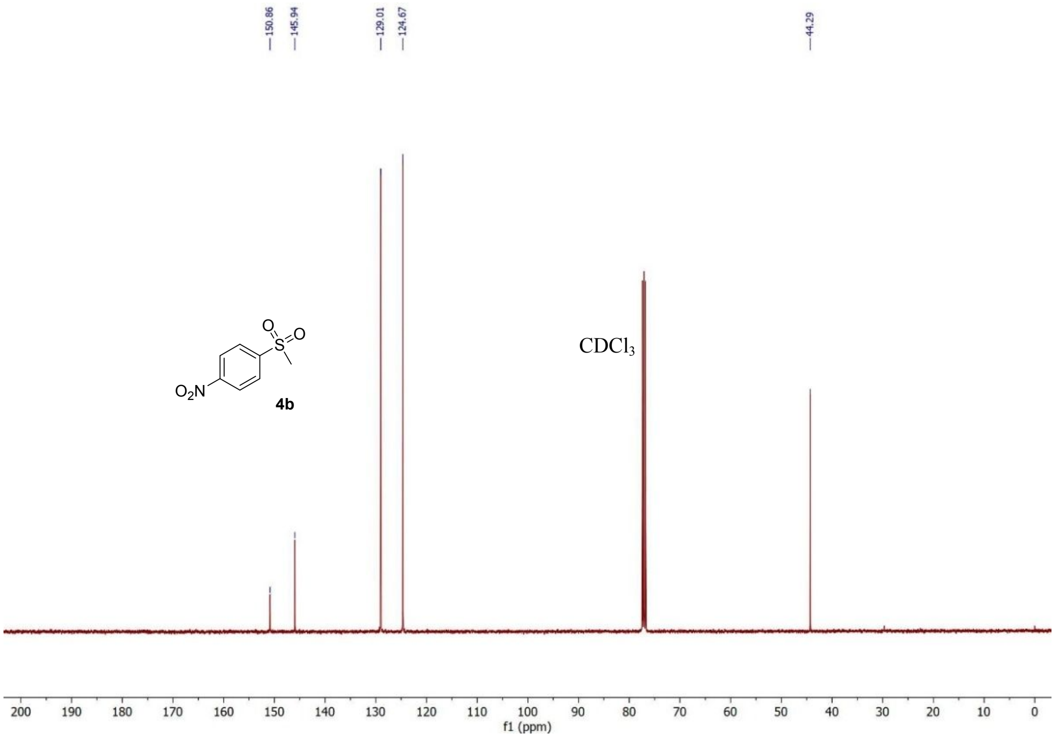

^{1}H NMR (400 MHz)

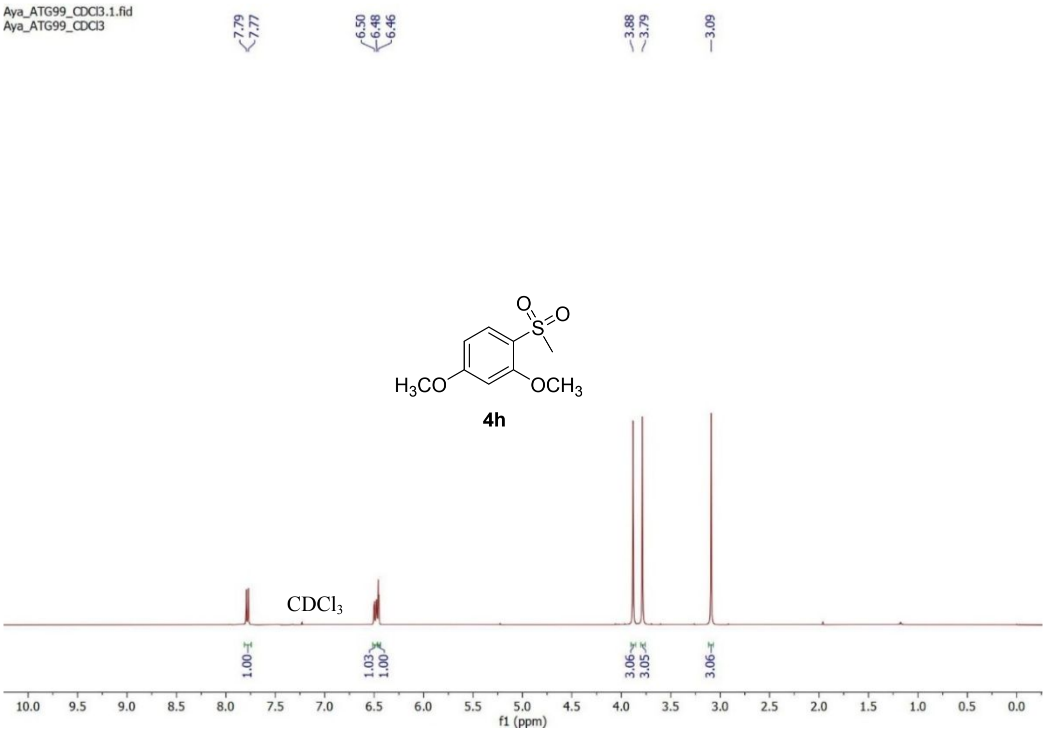

^{13}C-NMR (100 MHz)

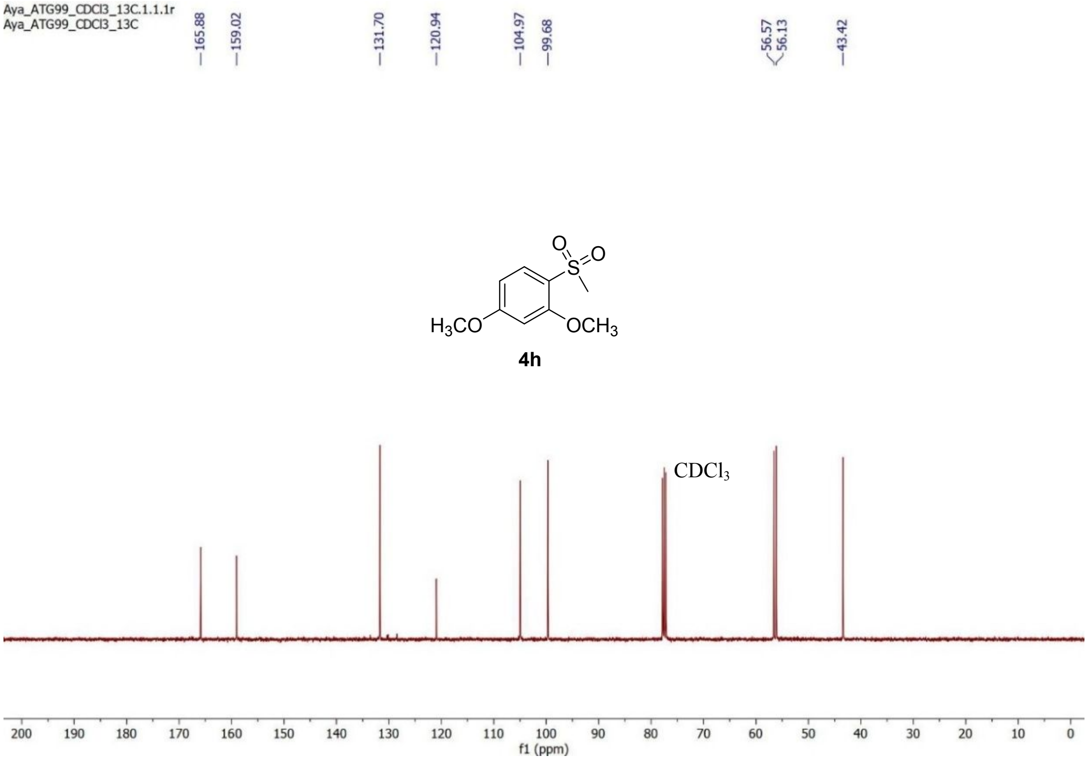

^{1}H NMR (400 MHz)

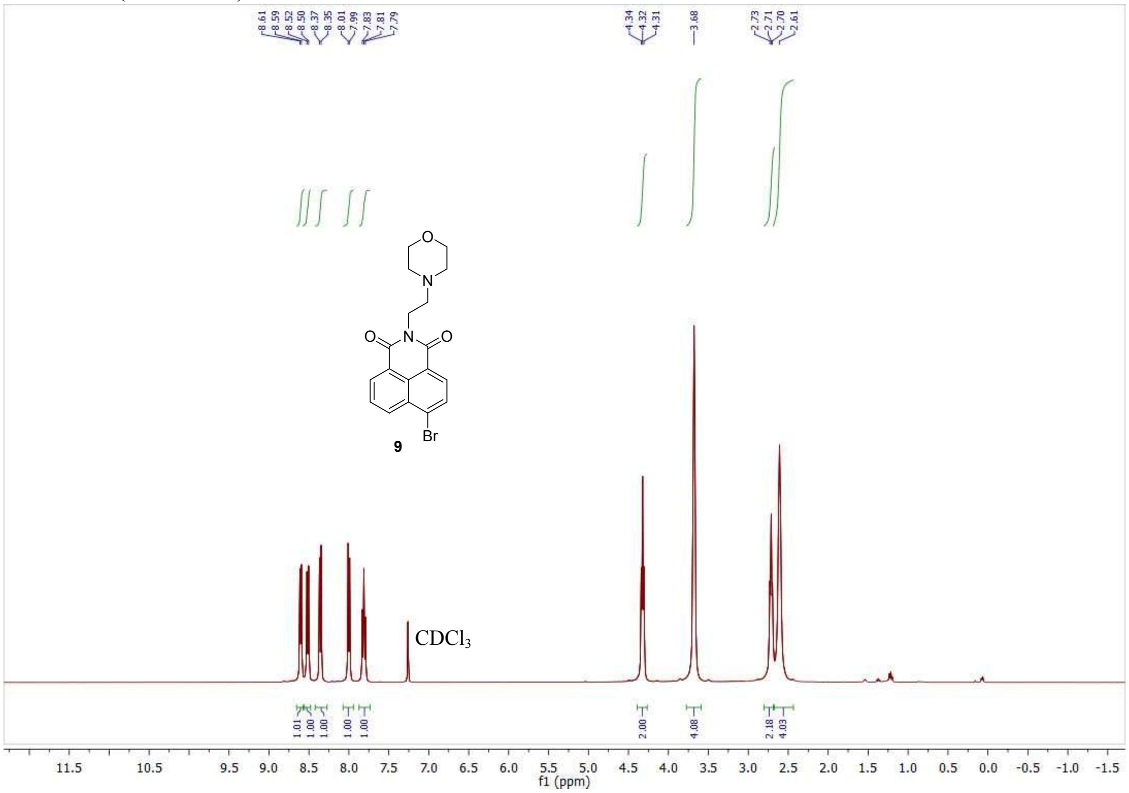

^{1}H NMR (400 MHz)

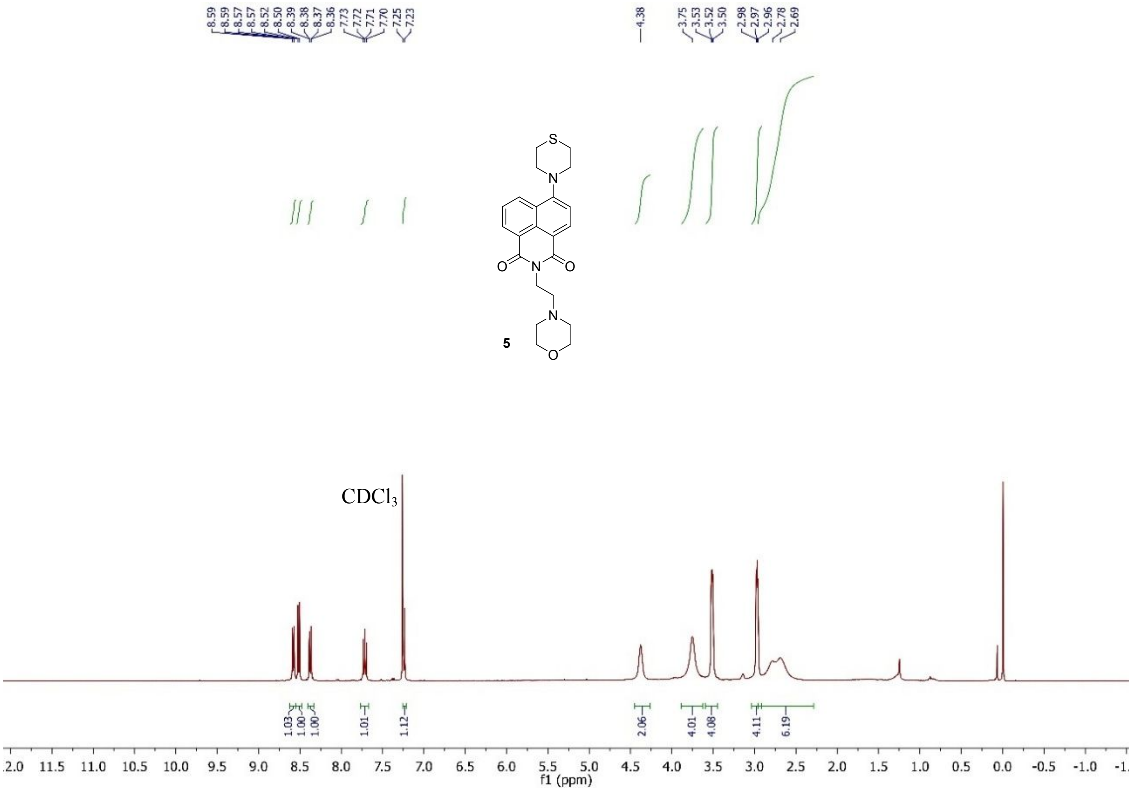

HRMS

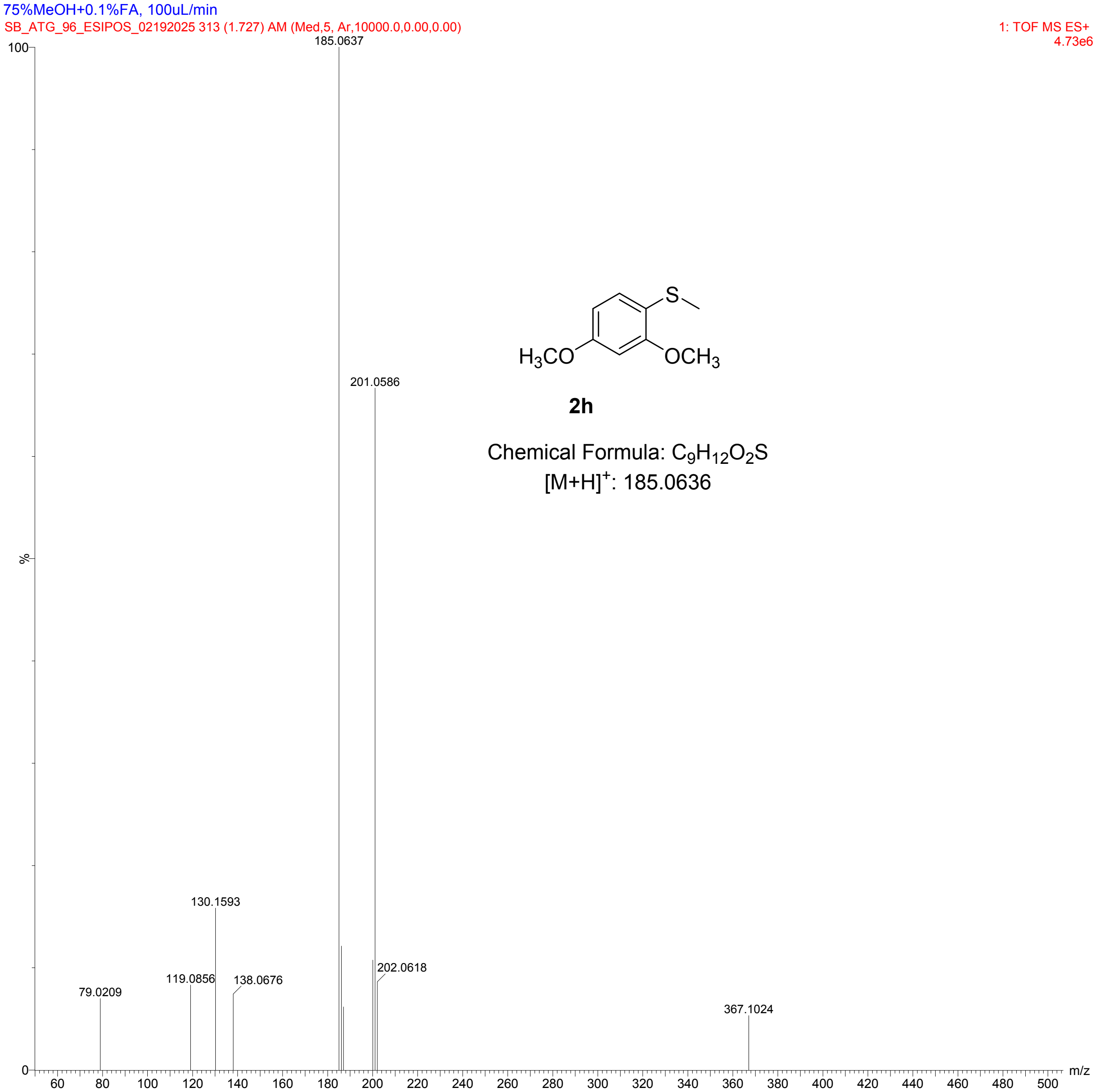

2h

Chemical Formula: $C_9H_{12}O_2S$

$[M+H]^+$: 185.0636

HRMS

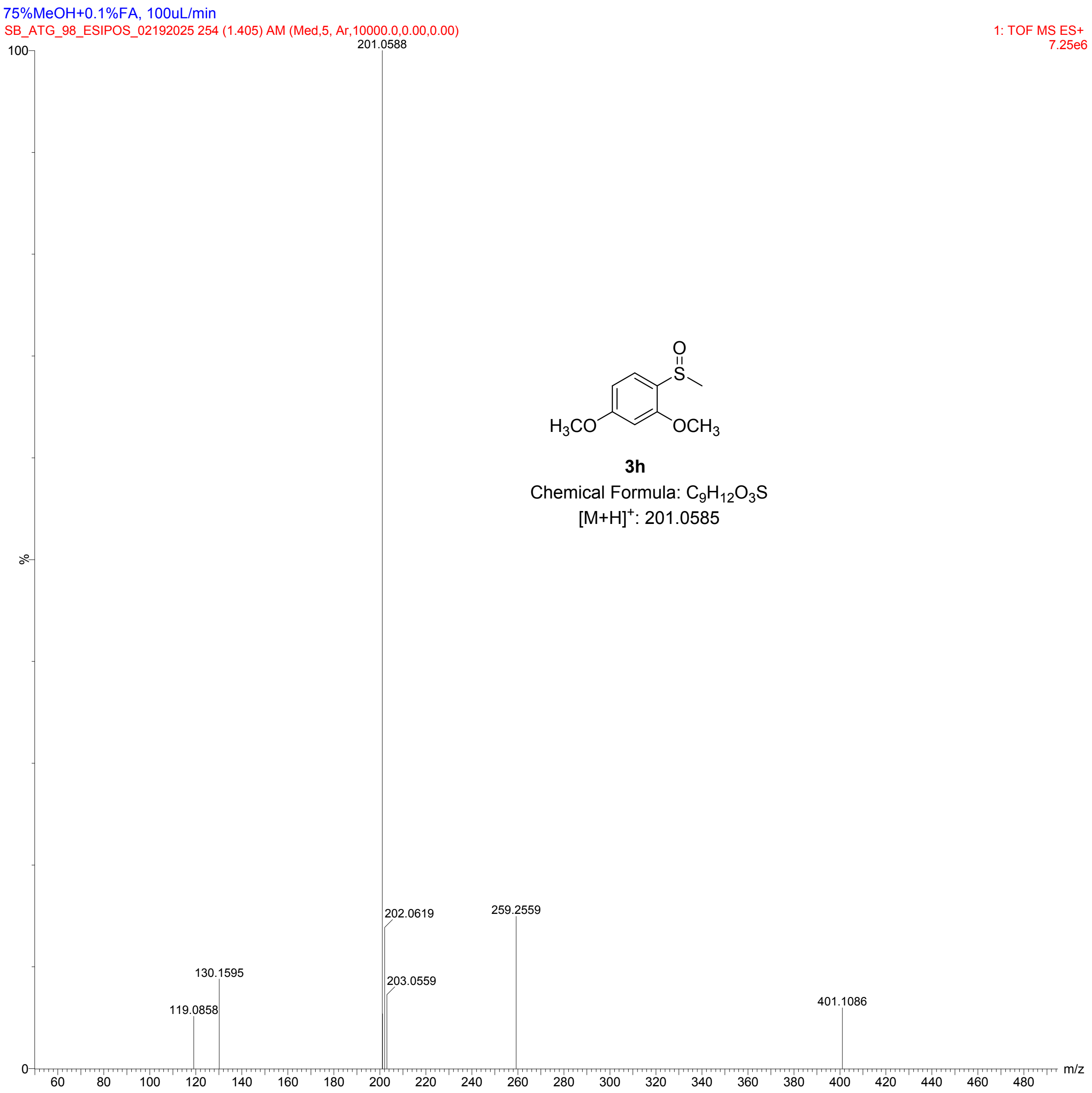
75%MeOH+0.1%FA, 100uL/min
SB_ATG_98_ESIPOS_02192025 254 (1.405) AM (Med,5, Ar,10000.0,0.00,0.00)
1: TOF MS ES+
7.25e6
201.0588
202.0619
259.2559
130.1595
203.0559
119.0858
401.1086
%
m/z
3h
Chemical Formula: C9H12O3S
[M+H]+: 201.0585

HRMS

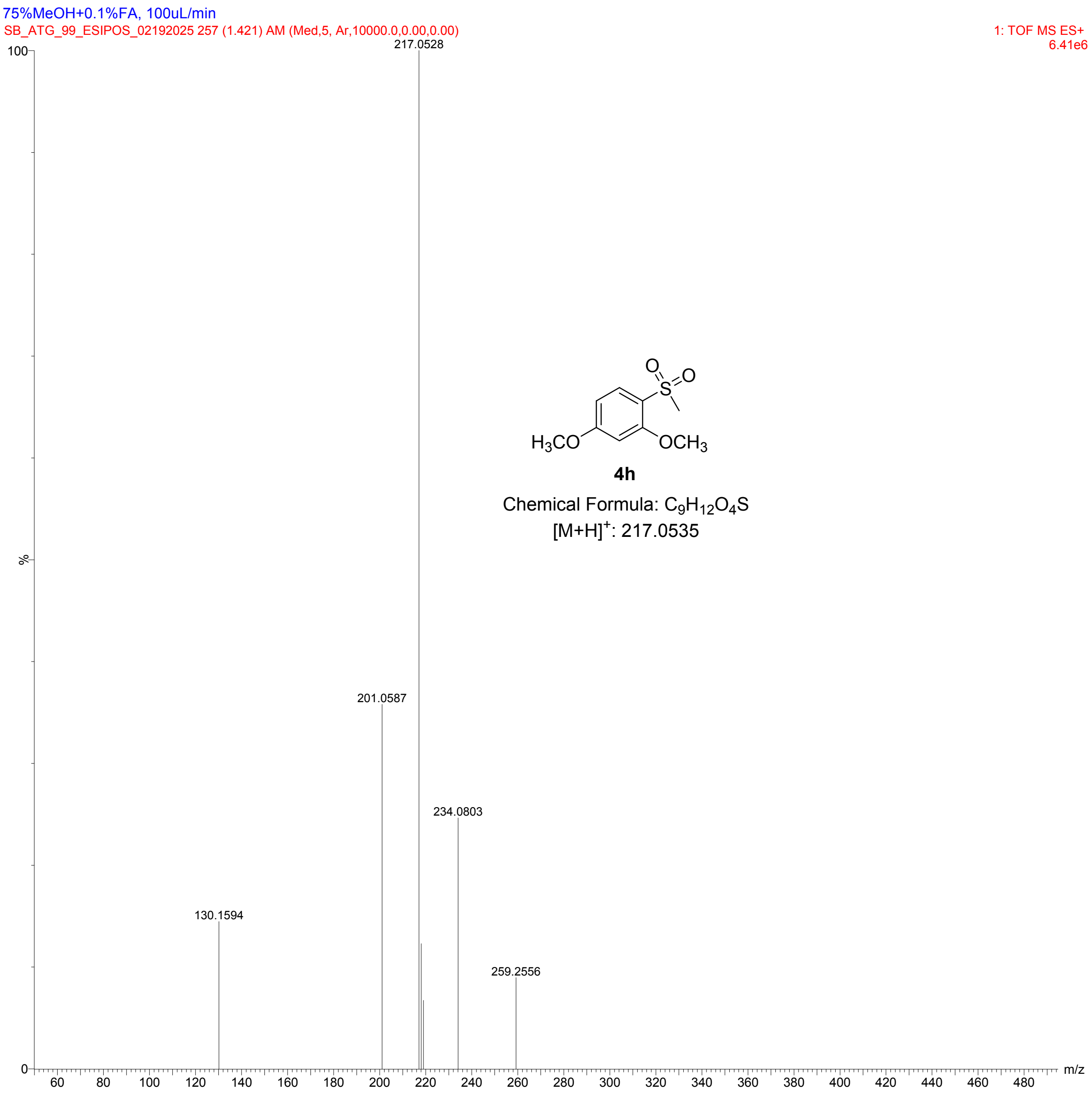

75%MeOH+0.1%FA, 100uL/min
SB_ATG_99_ESIPOS_02192025 257 (1.421) AM (Med,5, Ar,10000.0,0.00,0.00)
1: TOF MS ES+
6.41e6
217.0528
201.0587
234.0803
130.1594
259.2556
%
m/z
4h
Chemical Formula: C9H12O4S
[M+H]+: 217.0535

References

(1) Bera, P. K.; Kumari, P.; Abdi, S. H. R.; Khan, N. U. H.; Kureshy, R. I.; Subramanian, P. S.; Bajaj, H. C. *In situ*-generated chiral iron complex as efficient catalyst for enantioselective sulfoxidation using aqueous H2O2 as oxidant. *Rsc Adv* **2014**, *4* (106), 61550-61556.
(2) Kerr, W. J.; Reid, M.; Tuttle, T. Iridium-Catalyzed C-H Activation and Deuteration of Primary Sulfonamides: An Experimental and Computational Study. *Acs Catal* **2015**, *5* (1), 402-410.
(3) Liu, J.; Li, J.; Ren, J. M.; Zeng, B. B. Oxidation of aromatic amines into nitroarenes with CPBA. *Tetrahedron Lett* **2014**, *55* (9), 1581-1584.
(4) Liu, T.; Xu, Z.; Spring, D. R.; Cui, J. A lysosome-targetable fluorescent probe for imaging hydrogen sulfide in living cells. *Org Lett* **2013**, *15* (9), 2310-2313.
(5) Li, N.; Tan, Q. Y.; Wang, Q.; Huang, K.; Yang, X. L.; Liang, L. J. A naphthalimido-based fluorescent probe for HOCl sensing with excellent lysosome-targeted performance. *J Mol Struct* **2025**, *1329*, 141393.
